# Plant-Derived Compounds as Potential Sensitizers to Immunotherapy in Melanoma

**DOI:** 10.3390/ijms27104423

**Published:** 2026-05-15

**Authors:** Oana Bătrîna, Roxana Negrea-Ghiulai, Liana Suciu, Roxana Racoviceanu, Elisabeta Atyim, Mihaela Jorgovan, Tamara Maksimovic, Alexandra Mioc, Cristina Trandafirescu, Codruța Șoica, Marius Mioc

**Affiliations:** 1 Faculty of Pharmacy, “Victor Babeș” University of Medicine and Pharmacy, 2 Eftimie Murgu Square, 300041 Timisoara, Romania; oana.esanu@umft.ro (O.B.); roxana.ghiulai@umft.ro (R.N.-G.); elisabeta.atyim@umft.ro (E.A.); mihaela.coban@umft.ro (M.J.); tamara.maksimovic@umft.ro (T.M.); alexandra.mioc@umft.ro (A.M.); trandafirescu.cristina@umft.ro (C.T.); codrutasoica@umft.ro (C.Ș.); marius.mioc@umft.ro (M.M.); 2 Research Centre for Experimental Pharmacology and Drug Design (X-Pharm Design), “Victor Babes” University of Medicine and Pharmacy, 2 Eftimie Murgu Square, 300041 Timisoara, Romania

**Keywords:** checkpoint modulation, melanoma, plant-derived compounds, immune activation, tumor microenvironment

## Abstract

Compounds of plant origin have increasingly emerged as anticancer agents through direct cytotoxicity and sensitizing mechanisms. Melanoma remains the most aggressive form of skin cancer that exhibits a steadily increasing number of new cases globally each year, thus urgently requiring more effective therapeutic strategies. Therefore, phytochemicals can be considered promising candidates, particularly when used in combination with immune checkpoint inhibitors. Their ability to optimize therapeutic efficacy and strengthen antitumor immune responses is mediated through various mechanisms that include the stimulation of T cell activity, the regulation of the TME, the activation of intrinsic immune responses and cytokine signaling, and the regulation of immune checkpoints such as PD-1/PD-L1, CTLA-4, and LAG-3. Additionally, these compounds can alter key signaling pathways that control immune regulation. Nevertheless, the extrapolation of preclinical studies to clinical applications remains limited by insufficient clinical evidence, the lack of standardized therapeutic protocols, and poor pharmacokinetic behavior. Consequently, further studies are required in order to clarify their actual efficacy and to better define their role in modern oncology. This article aims to review the mechanisms that underlie the anticancer sensitizing activity of major classes of plant-derived compounds such as polyphenols, flavonoids, terpenoids, alkaloids, and isothiocyanates. The available preclinical and clinical evidence were reported together with their potential synergistic effects when combined with immune checkpoint inhibitors. An important aspect related to the anticancer effects of these compounds lies in their ability to simultaneously target multiple signaling pathways. Furthermore, advanced formulations such as nanoparticulated delivery systems are discussed as strategies to optimize their clinical application and therapeutic outcomes.

## 1. Introduction

Melanoma is one of the most aggressive human malignancies that, despite representing a small fraction of total skin cancer cases, contribute hugely to skin cancer mortality. Globally, melanoma accounts for 1.7% of global cancer diagnoses [1]. Unfortunately, its incidence worldwide continues to increase, particularly in fair-skinned populations as well as in regions with high ultraviolet exposure. A large international analysis revealed a significant amount of new cases every year and predicted further increases until 2040 [2]. Epidemiologically, the main factors favoring melanoma development are intense exposure to UV radiation, personal history of sunburn or phenotypic susceptibility, as well as older age. In terms of survival, melanoma remains strongly dependent on its stage of diagnosis, with total surgical cure for early localized diseases but major clinical challenges in advanced diseases [3].

Advanced melanoma is characterized by biological complexity due to its molecular and clinical heterogeneity, which makes its management highly challenging [4]. Although the discovery of key oncogenic triggers such as the Mitogen-Activated Protein Kinase (MAPK) pathway mutations, i.e., B-Rapidly Accelerated Fibrosarcoma (BRAF) and Neuroblastoma RAS viral oncogene homolog (NRAS), have enabled the development of targeted therapies [5], response to treatment is frequently limited by rapidly emerging resistance mechanisms that include the pathway reactivation or the development of alternative signaling pathways [6]. Additionally, melanoma exhibits the tendency to disseminate early, frequently triggering brain metastasis that worsens its prognosis, limits drug therapeutic efficacy and, overall, makes treatment decisions more difficult [7]. Despite achieving major progress in systemic therapy, significant challenges remain, such as the need to identify reliable biomarkers to monitor cancer evolution, the optimization of drug combinations and treatment sequencing, as well as the identification of improved strategies to address less common melanoma subtypes or resistant types of the disease [8,9].

Melanoma therapy has seen a major breakthrough with the introduction of immune checkpoint inhibitors that were able to change the once very limited long-term survival into efficient tumor control and prolong overall survival (OS) in a significant number of patients. For example, ipilimumab activates the immune system by inhibiting Cytotoxic T-Lymphocyte Antigen 4 (CTLA-4), a protein receptor that downregulates the immune system [10]; its therapeutic outcome was surpassed by newer therapies involving combined Programmed Death-1 (PD-1)/CTLA-4 inhibitors, such as nivolumab/pembrolizumab and ipilimumab, that are able to synergistically boost the immune system, albeit at the cost of higher systemic toxicity [11,12]. More recently, the introduction of dual Lymphocyte Activation Gene 3 (LAG-3)/PD-1 inhibitors has contributed to further improved therapeutic outcomes [13]. Such agents hold significant clinical relevance due to their ability to not only improve the therapeutic response and the OS but also trigger long-term immune-mediated disease control in metastatic melanoma, where selected patients (up to 50%) were able to experience durable remission instead of mere transient palliation [14,15].

However, the limitations of such therapies remain considerable, with a large number of patients exhibiting primary or acquired resistance due to the tumor’s intrinsic escape mechanisms, reduced antigen production, impaired interferon signaling, inadequate anti-tumor T-cell effector function, or a particular tumor microenvironment (TME) [14,16]. Another issue is the occurrence of immune-related adverse effects, particularly for combination regimens, that may involve multiple organs, persist in the long term even after treatment discontinuation, and even become life-threatening [17,18]. Moreover, there are currently no highly sensitive and specific predictive biomarkers for treatment monitoring, although several categories have been investigated (i.e., Programmed Death-Ligand 1 (PD-L1) expression, immune infiltrates, mutation burden genes, peripheral blood constituents, etc.) [19]. Thus, despite their crucial role in melanoma management, immune checkpoint inhibitors remain constrained by insufficient therapeutic response, resistance development, systemic toxicity, and inadequate monitoring ability, all of which make melanoma a growing public health issue.

Plant-derived compounds are currently under investigation as adjuvants in melanoma treatment due to their chemically diverse and biologically active scaffolds that have been historically involved in the anticancer therapy; such compounds have been revealed to be able to modulate several hallmarks of tumor progression such as cell proliferation, apoptosis, tumor angiogenesis and metastasis, as well as the inflammatory signaling pathways [20,21]. This multitargeted behavior can be particularly of use against melanoma due to its previously mentioned pathway bypass, phenotypic plasticity, and rapidly developing resistance mechanisms. The literature has reported several phytocompounds able to enhance the anticancer activity of conventional drugs; for example, schweinfurthin compounds improved the therapeutic outcome of associated anti-PD-1 inhibitors in murine melanoma [22], while the protocatechuic aldehyde exhibited synergistic activity with dacarbazine in melanoma cells, where they increased DNA damage and apoptosis [23]. Compounds such as quercetin [24] and curcumin [25] were found to alter melanoma-specific pathways and are therefore considered as active drugs in combination regimens. However, this approach poses challenges in terms of preclinical-to-clinical extrapolation: most studies cannot surpass the preclinical stage due to poor aqueous solubility, low bioavailability, high drug metabolism rate, or formulation issues, therefore hindering clinical validation [26,27].

There are several reviews that focus on the use of plant-derived compounds against melanoma, but only a few address the specific mechanisms of immunotherapy. Previous reviews have been mainly tackling the antiproliferative, pro-apoptotic, anti-invasive, and chemopreventive effects of phytochemicals, without discussion or with minimal discussion of how such compounds are able to influence the immune-based treatments [26,28,29]. A more specific review was published by Tabolacci et al. in 2023 who focused on the immunomodulatory activity of phytochemicals in melanoma, emphasizing their effects on cytokines and inflammation pathways but limiting their observations at the preclinical level without addressing the sensitization issues of already approved checkpoint inhibitors [30]. Conversely, other reviews indeed approached the activity of phytocompounds as enhancers of cancer immunotherapy, not specifically in melanoma but across many tumor types as well [31,32]. A very recent systematic review focused directly on natural compounds as immune checkpoint modulators and identified 19 preclinical studies of compounds able to exhibit PD-1/PD-L1, CTLA-4, and LAG-3-related mechanisms [33]. Of note, previous reviews have not systematically considered melanoma biology, primary or acquired resistance towards the checkpoint blockade, or the mechanism of action of phytocompounds; moreover, there are no reviews that specifically focus on phytocompounds as sensitization agents in melanoma treatments. Therefore, there is a clear need for a study that focuses on phytocompounds’ potential to sensitize anti-melanoma immunotherapy and identify the specific requirements needed for their clinical development.

The current review aims to challenge the current paradigm that considers phytocompounds only as direct anticancer agents through various mechanisms and instead suggests an orientation toward their role as sensitizers of melanoma immunotherapy. It emphasizes their ability to surpass both primary and acquired drug resistance by the modulation of the TME, the increase in tumor immunogenicity, as well as the restoration of the antitumor immune responses. Moreover, the study aimed to search emerging evidence regarding their synergistic effects with immune checkpoint inhibitors in order to identify promising adjuvants able to enhance the therapeutic efficacy of such drugs and to extend the benefits of immunotherapy to a larger patient population. Non-melanoma evidence was introduced only when it provided mechanistic context relevant to immune-responsive tumors and was not used as proof of efficacy in melanoma.

## 2. Melanoma Immunotherapy Overview

### 2.1. Immune Checkpoints in Melanoma

T-cells or lymphocytes T are key components of the adaptive immune system and, along with lymphocytes B or B-cells, account for 20–40% of all white blood cells (WBCs). They originate in the bone marrow, mature in the thymus, and are finally located in the lymph nodes or spleen. Unlike B-cells that are able to produce antibodies, T cells act by fighting infected or cancerous cells. T cells can perform different roles in immune response, such as (i) cytotoxic T cells/CD8+ cells (CD8+ receptor) that kill infected/cancer cells, (ii) helper T cells/CD4+ cells (CD4+ receptor) that communicate with other cells of the immune system and send attack signals against an intruder, or (iii) regulatory T cells (Treg) that inhibit the over activation of the immune responses and prevent the onset of autoimmune diseases. During the first step of T-cell activation (signal 1), antigen-presenting cells (APCs) present antigen peptides via the major histocompatibility complex II (MHC II) followed by the recognition and binding of T cells via the T-cell receptor (TCR). The next step (signal 2/co-stimulation) consists of the binding between cluster of differentiation 28 (CD28, a constitutively expressed receptor on T cells) and B7-1/CD80 or B7-2/CD86 (upregulated molecules on the APC during inflammation). The results of these interactions activate the Phosphoinositide 3-Kinase (PI3K)/RAC (Rho family)-alpha serine/threonine-protein kinase (AKT) pathway that increases Interleukin-2 (IL-2) production, promotes T-cell proliferation and increases cytokine production that guides T-cell differentiation into effector cytotoxic T lymphocytes (CTLs) and survival, briefly fully activating T cells (signal 3) [34,35].

**Figure 1:**
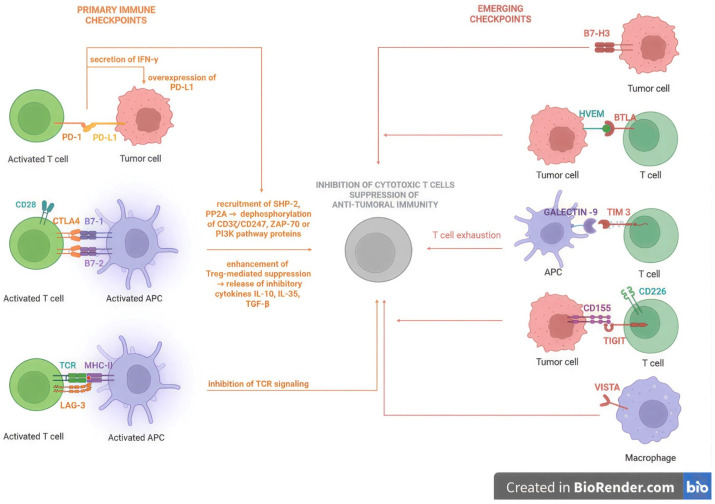
Immune system inhibitory regulatory pathways (immune checkpoints) (Created in BioRender. Oana Bătrîna. (2026) https://BioRender.com/0lawliw).

Immune checkpoints or immune brakes are immune system inhibitory regulatory pathways that prevent collateral tissue damage during immune responses and preserve self-tolerance. As a result, immune cells, especially T cells, receive negative signals through receptor–ligand interactions (Figure 1) [36].

**Primary immune checkpoints in melanoma**

**CTLA-4/CD152** is a receptor expressed on activated T cells/Tregs, similar to CD28, upregulated after T-cell activation. CTLA-4 competes with CD28 with higher affinity for B7 (ligands/proteins on APCs) molecules, delivering an inhibitory signal that prevents both the proliferation and activation of CTLs mainly in the lymph nodes as well as IL-2 production [37]. The core mechanism relies on the recruitment of phosphatases, primarily Src homology region 2-containing phosphatase-2 (SHP-2) and, to a lesser extent, protein phosphatase 2A, that are able to dephosphorylate signaling molecules such as CD3ζ/CD247 (transmembrane protein subunit of TCR), Zeta-chain-associated protein kinase 70 (ZAP-70), or CD28-associated signaling proteins (PI3K pathway). Another mechanism implicates the enhancement of Treg-mediated suppression that in turn stimulates the release of inhibitory cytokines such as IL-10, IL-35, and Transforming Growth Factor-β (TGF-β). In melanoma, CTLA-4 prevents the priming and activation of cytotoxic T cells while Tregs suppress antitumoral immunity, resulting in an impaired initiation of anti-melanoma immune response [38].

**PD-1/(CD279)** is another inhibitory receptor expressed on activated T cells/B cells/natural killer (NK) cells that, after engagement, reduces T-cell activity by the recruitment of SHP-2 phosphatase. In turn, it leads to the dephosphorylation of key signaling molecules and prevents the overactivation of the immune response mainly in peripheral tissues, in a similar way following CTLA-4 activation [39].

**PD-L1** is a transmembrane protein, a ligand expressed on tumor cells such as melanoma cells, that binds to PD-1 receptor, disabling the immune response. Upon T cells’ contact with melanoma cells, they secrete interferon-γ (IFN-γ), which turn causes melanoma cells to overexpress PD-L1 and lead to inhibition of T-cell activity via the PD-1 receptor. This mechanism is a vicious cycle by which melanoma cells manipulate the immune cells and prevent their attack while leading to T-cell exhaustion and inefficiency [40].

**LAG-3** is an inhibitory immune checkpoint receptor that downregulates T-cell activation and function. It has structural similarities to CD4 because both genes are located on chromosome 12 and roughly 20% of the amino acids are identical. LAG-3 binds to MHC II molecules and sends an inhibitory signal. This makes the immune system less active, impairs T-cell activation and growth, and stops TCR signaling. LAG-3 is overexpressed in melanoma, which lets tumor cells avoid the immune system. As a result, T-cell function is impaired and the tumors gain aggressiveness. LAG-3 and PD-1 are typically found on the same T cells, which makes the immune system less effective and T cells less active. Additionally, LAG-3 and CTLA-4 alter TCR signaling pathways, thus enhancing immunological tolerance and inhibiting T-cell activation [41,42].

**Emerging checkpoints in melanoma**

The emerging immune checkpoints are a new class of inhibitory pathways that include T-cell immunoglobulin and mucin domain-containing protein 3 (TIM-3), T-cell immunoreceptor with immunoglobulin and ITIM domain (TIGIT), V-domain Ig suppressor of T-cell activation (VISTA), CD276 (B7-H3), and B and T lymphocyte attenuator (BTLA) and play a critical role in melanoma immune evasion and resistance to immunotherapy. They are often expressed with PD-1 and LAG-3; thus, current therapeutic strategies target multiple inhibitory pathways to overcome resistance and improve the treatment outcome.

**TIM-3** is a receptor found on exhausted T cells and NK cells. It interacts with different ligands, such as galectin-9, and contributes to the final exhaustion of T cells [43].

**TIGIT** is a receptor found on T cells and NK cells. TIGIT inhibits T-cell activation and NK cell cytotoxicity by competing with the CD226 co-stimulatory receptor for binding to the CD155 ligand, which is upregulated on melanoma cells, thus promoting immune suppression within the TME [44].

**VISTA** is expressed mainly on macrophages and monocytes and suppresses early T-cell activation [45].

**B7-H3** is an immunoregulatory protein overexpressed in melanoma cells associated with inhibition of T-cell activity and increased tumor progression and metastasis [46].

**BTLA** is an inhibitory immune checkpoint receptor that binds to the herpes virus entry mediator on tumor cells and promotes immune suppression and T-cell inhibition [47].

### 2.2. Current Immunotherapies in Melanoma

**Figure 2:**
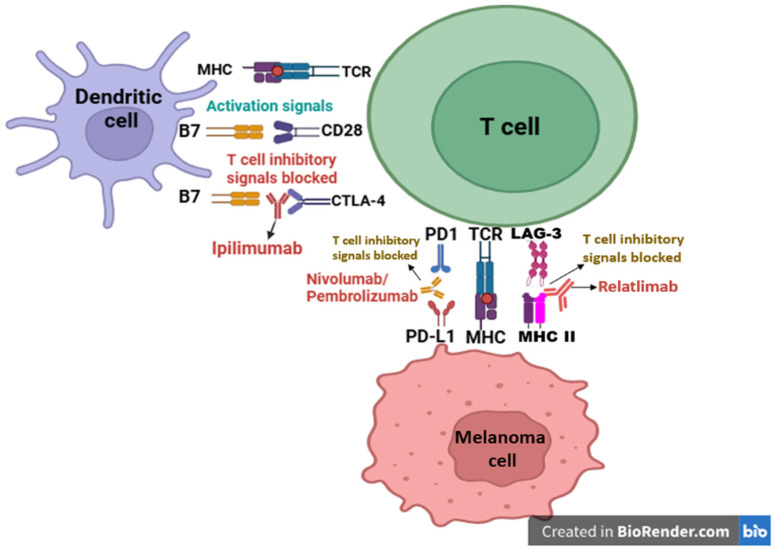
The mechanism of action of immune checkpoint inhibitors (Created in BioRender. Oana Bătrîna. (2026) https://BioRender.com/dvc1d5n).

Current immunotherapies in melanoma are classified as checkpoint inhibitors and include anti-PD-1 (e.g., nivolumab, pembrolizumab), anti-CTLA-4 (e.g., ipilimumab), and anti-LAG-3. As a consequence of checkpoint blockade, T cells regain activity and are thus able to attack melanoma cells (Figure 2).

**Pembrolizumab** is a humanized IgG4 monoclonal antibody that targets PD-1 and restores TCR and CD28 signaling by preventing PD-1’s interaction with PD-L1 and PD-L2. This boosts antitumor immunity instead of directly destroying tumor cells [10]. In KEYNOTE-006, pembrolizumab significantly enhanced OS, progression-free survival (PFS), and objective response rate (ORR ~30–40%) in comparison to ipilimumab [48]. Long-term follow-up confirmed the durability of the outcome. At 7 years, the median OS remained at 32.7 months with pembrolizumab versus 15.9 months with ipilimumab, and 7-year OS was 37.8% versus 25.3% [49]. According to the Phase III KEYNOTE-006 study, the 10-year follow-up further confirmed a persistent long-term survival benefit and supports pembrolizumab as a durable standard-of-care option in advanced melanoma [50].

These conclusions are corroborated by real-world data. A substantial population-based analysis indicated a median OS of approximately 27 months and a 2-year OS rate of 52%, aligning with clinical trial findings; however, outcomes were inferior in older patients [51]. Significantly, pembrolizumab exhibits unusual response patterns. In a Danish study, over 22% of patients originally presented with stable disease, and around 40% of these subsequently attained objective responses [52]. This underscores that delayed responses are prevalent and that early stable disease does not inherently signify treatment failure.

Despite its efficacy, response rates are still low (around 30–40%), which shows that the tumor is able to avoid the immune system, e.g., by poor antigen presentation, defective interferon-γ signaling, limited T-cell infiltration and immunosuppressive TMEs [53]. Resistance can be innate or developed. Primary resistance is linked to insufficient immune activation, while acquired resistance may result from genetic changes, including Janus kinase (JAK)1/JAK2 mutations that interfere with interferon signaling or the loss of β2-microglobulin that hinders antigen presentation [54].

Pembrolizumab is usually better tolerated than CTLA-4 inhibitors, although it can cause immune-related adverse events (irAEs) since it activates the immune system in a way that is not specific. Fatigue, diarrhea, and rash are common side effects. More serious irAEs can impact the gastrointestinal tract, liver, lungs, endocrine organs, and other systems [55].

**Nivolumab** is a fully human IgG4 monoclonal antibody that targets PD-1. Nivolumab does not destroy melanoma cells directly, but it instead blocks the connection between PD-1 and PD-L1 and PD-L2, which stops inhibitory signaling on activated T cells and restores anticancer immune function.

During the CheckMate 066 trial, nivolumab was first shown to be efficient in patients with untreated advanced melanoma who did not have a BRAF mutation. It significantly increased OS, PFS, and ORR compared to dacarbazine. The trial showed that nivolumab had 40.0% ORR compared to 13.9% for dacarbazine and that the OS rate after one year was 72.9% compared to 42.1% for dacarbazine [56]. The CheckMate 067 trial demonstrated the long-term clinical implications of nivolumab. Monotherapy had a 44% 5-year survival rate, nivolumab with ipilimumab had a 52% survival rate, and ipilimumab alone had a 26% survival rate [11,57]. The last report, after 10 years of follow-up, demonstrated that the long-term benefits were still present: the median melanoma-specific survival was 49.4 months with nivolumab monotherapy and more than 120 months with nivolumab + ipilimumab, compared to 21.9 months with ipilimumab. These data confirmed nivolumab-based therapy as one of the most enduring systemic treatments for advanced melanoma [15].

Nivolumab also plays a significant role in other melanoma contexts. In individuals with resected high-risk melanoma, the CheckMate 238 study demonstrated prolonged recurrence-free and distant metastasis-free survival benefits for adjuvant nivolumab compared to ipilimumab, with a more favorable safety profile. Nivolumab plus ipilimumab showed clinically significant efficacy in untreated melanoma brain metastases, solidifying checkpoint inhibition as a crucial approach even in intracranial illness [58]. Even with these improvements, nivolumab monotherapy has limited response rates, usually just about one-third to two-fifths of patients. This implies that many patients either do not respond at all or only obtain modest benefits. This restricted response is due to a number of immune-evasion mechanisms, including poor antigen presentation, faulty interferon-γ signaling, inadequate T-cell priming or infiltration, and TMEs that suppress immunological responses [56]. Resistance to nivolumab may be either primary or acquired. Primary resistance is typically linked to noninflamed or “cold” tumors, impaired antigen presentation, stromal or myeloid exclusion of T cells, and inadequate baseline immunological activation. Acquired resistance following an initial response may arise from genetic modifications that hinder tumor detection or interfere with interferon responsiveness, typically including JAK1/JAK2 pathway abnormalities and β2-microglobulin loss, which compromise MHC I antigen presentation. These mechanisms elucidate the reasons behind the occurrence of lasting remissions in certain patients, whereas others experience relapse following initial improvement [54,59,60].

Nivolumab is usually better tolerated than CTLA-4 blocking, but it can cause irAEs since it activates the immune system in a nonspecific way. Common side effects include tiredness, rashes, itching, diarrhea, and nausea. More serious immunological side effects might affect the heart, liver, lungs, endocrine glands, kidneys, brain system, and gastrointestinal tract [61,62].

**Ipilimumab** is a fully human IgG1 monoclonal antibody that promotes T-cell priming and proliferation by inhibiting CTLA-4, especially within lymphoid tissues, enhancing its antitumor immunity instead of directly causing melanoma cell death. The biological justification for ipilimumab in melanoma stems from the tumor’s significant immunogenicity. While CTLA-4 blockage can stimulate antitumor immunity, it operates at a more proximal stage in the immune response and typically exhibits worse selectivity than PD-1 blocking, which partially accounts for its sustained efficacy in certain individuals and its comparably elevated safety profile [63].

In contemporary melanoma treatment, ipilimumab has historical and mechanistic significance; nevertheless, its function as a monotherapy has diminished due to the superior efficacy and tolerability of anti-PD-1 therapies and PD-1-based combos, as revealed by the above-mentioned CheckMate 067 trial [57]. Ipilimumab has also exhibited efficacy in the adjuvant context. In the EORTC 18071 trial, adjuvant ipilimumab markedly enhanced recurrence-free survival following full resection of high-risk stage III melanoma compared to placebo. Extended follow-up demonstrated that recurrence-free survival, distant metastasis-free survival, and OS all favored ipilimumab, thereby affirming its genuine anticancer activity. Nonetheless, these advantages were accompanied by significant toxicity, resulting in elevated rates of treatment termination [64,65].

The shortcomings of ipilimumab monotherapy were further elucidated in the CheckMate 238 trial, which contrasted adjuvant nivolumab with adjuvant ipilimumab in resected stage IIIB-C/IV melanoma. Nivolumab demonstrated superior recurrence-free survival and a significantly more acceptable safety profile [66]. These results elucidate the constraints of ipilimumab’s present clinical application as monotherapy: although it may yield sustained benefits in a minority of patients, the ORR and long-term outcomes are typically subpar compared to PD-1-based therapies. In practice, the low response rates to ipilimumab indicate various mechanisms of tumor immune evasion, such as inadequate antigen presentation, insufficient T-cell infiltration, immunosuppressive myeloid and regulatory cell populations, and compensatory inhibitory pathways that are not entirely mitigated by CTLA-4 blockade alone [57].

Resistance to ipilimumab may be classified as either primary or acquired, although the data on resistance is less molecularly defined for CTLA-4 inhibition compared to PD-1 drugs. Primary resistance is typically linked to inadequate baseline immune priming, diminished tumor immunogenicity, and inhibitory TMEs. Clinically, a prominent contemporary observation is that following resistance to anti–PD-1 therapy, ipilimumab alone demonstrates little efficacy, whereas the combination of ipilimumab with anti–PD-1 shows greater effectiveness. In the multicenter research conducted by da Silva et al., the combination demonstrated a superior ORR, extended PFS, and prolonged OS compared to ipilimumab monotherapy following anti–PD-(L)1 resistance, while exhibiting a comparable incidence of grade 3–5 toxicity [67].

The pharmacotoxicology of ipilimumab is a particular clinical feature. Due to its broad amplification of immunological activation through CTLA-4 inhibition, ipilimumab is linked to a high incidence of irAEs affecting the skin, gastrointestinal tract, liver, endocrine organs, and other systems. In adjuvant melanoma, the burden of adverse events has been notably significant: the EORTC 18071 trial indicated that the adverse-event profile aligned with that of metastatic melanoma yet exhibited elevated rates of severe toxicities, especially endocrinopathies. The 5-year analysis revealed that treatment discontinuation due to adverse events occurred in 53.3% of ipilimumab-treated patients compared to 4.6% in the placebo group [65].

**Relatlimab** is a human IgG4 monoclonal antibody oriented to LAG-3 located on exhausted and activated T cells, acting as an immune checkpoint inhibitor. In melanoma, relatlimab is utilized clinically in conjunction with nivolumab as the fixed-dose formulation OPDUALAG^®^. The combination of LAG-3 inhibition with PD-1 blockade by nivolumab aims to counteract complementary inhibitory mechanisms that diminish antitumor T-cell activity [68].

The pivotal phase II/III RELATIVITY-047 trial disclosed the clinical significance of relatlimab in advanced melanoma. In previously untreated unresectable or metastatic melanoma, the combination of nivolumab and relatlimab enhanced PFS compared to nivolumab monotherapy in the initial report. The latest update on OS and response indicates that the median PFS was 10.2 months for nivolumab combined with relatlimab, compared to 4.6 months for nivolumab alone. The median OS was 51.0 months versus 34.1 months, respectively. The ORR for the combined therapy was 43.7%, whereas for nivolumab monotherapy it was 33.7% [69].

As for long-term activity, relatlimab can be considered an intermediate option between PD-1 monotherapy and PD-1/CTLA-4 combination therapy. During the three-year analysis conducted in 2025, a lasting efficacy was confirmed, indicating three-year PFS rates of 31.8% for the combination of nivolumab and relatlimab and 26.9% for nivolumab monotherapy, with corresponding three-year OS rates of 54.6% versus 48.0%, respectively. No new or unexpected safety concerns were observed. This combination is promising, as it offers enhanced efficacy compared to PD-1 monotherapy while reducing toxicity with respect to nivolumab plus ipilimumab [69].

Relatlimab demonstrates efficacy after prior PD-1/PD-L1 therapy; however, the response rates are significantly diminished in this context. In the RELATIVITY-020 trial, which included heavily pretreated patients with melanoma who had progressed on previous anti–PD-(L)1 therapies, the ORR was merely 12.0% in one cohort and 9.2% in another, with median PFS of 2.1 and 3.2 months, respectively; however, the responses that were observed could be enduring. This suggests that combination LAG-3/PD-1 blocking exhibits limited salvage efficacy following PD-1 failure; however, the majority of these patients remain unresponsive [70].

The limited response rates are attributable to LAG-3 being only one component of a more extensive resistance network. Mechanistic studies indicate that anti-LAG-3 combined with anti-PD-1 therapy alters the states of exhausted T cells and Tregs, while emerging resistance research suggests that unstable Treg programs, inadequate remodeling of dysfunctional T cells, and various microenvironmental constraints may affect therapeutic efficacy [71].

Relatlimab’s toxicity profile is a significant advantage compared to CTLA-4-based combo therapy. In the RELATIVITY-047 trial, grade 3–4 treatment-related adverse events were more prevalent with nivolumab + relatlimab compared to nivolumab alone; however, they remained significantly lower than those normally observed with nivolumab combined with ipilimumab. The FDA indicates prevalent adverse reactions, including musculoskeletal pain, fatigue, rash, pruritus, and diarrhea, and outlines significant immune-mediated toxicities such as pneumonitis, colitis, hepatitis, adrenal insufficiency, hypophysitis, thyroiditis, hyperthyroidism, hypothyroidism, nephritis, and rash [13].

A pertinent warning is that the advantages of relatlimab may not be uniformly applicable across various contexts. In the adjuvant RELATIVITY-098 study for resected stage III/IV melanoma, the combination of nivolumab and relatlimab did not enhance recurrence-free survival compared to nivolumab monotherapy. This unfavorable outcome indicates that the combination’s efficacy may be more contingent on context than previously anticipated and underscores that relatlimab’s most compelling evidence pertains to unresectable or metastatic untreated melanoma, rather than adjuvant escalation [72].

### 2.3. Resistance Mechanisms

**Figure 3:**
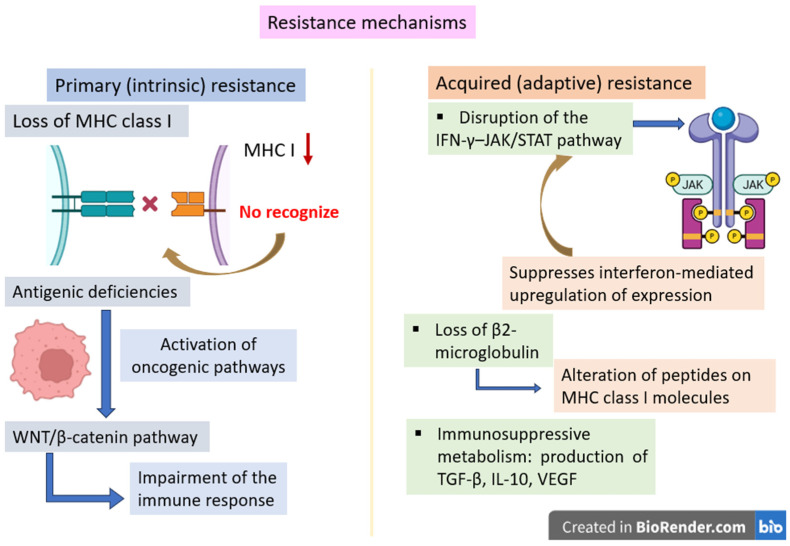
Immune evasion strategies in malignant melanoma: from primary to adaptive resistance. ↓—decrease/downregulation; Other arrows—leads to/generates (Created in BioRender. Oana Bătrîna. (2026) https://BioRender.com/rnnsdzw).

Malignant melanoma often eludes immunological suppression despite being a highly immunogenic tumor; this paradox can be explained by a series of immune evasion mechanisms that impair essential functions such as T-cell identification, activation, and effector activity. These resistance mechanisms can be classified as follows: (1) primary or intrinsic resistance, expressed before the treatment initiation, and (2) acquired or adaptive resistance, manifested after an initial response to therapy [73,74] (Figure 3).

Besides immune checkpoint activation, malignant melanoma cells display further mechanisms of tumor immune evasion, such as reduced visibility to the immune system by altering antigen presentation, like the downregulation or loss of MHC I expression, which generates compromised TCR recognition [73,75]. This mechanism is associated with primary resistance, because tumors that have an impaired antigen presentation are invisible to cytotoxic T cells [75].

Moreover, melanoma cells can develop errors in the antigen-processing system, such as mutations or loss of β2-microglobulin, a structural component of MHC I, which subsequently will alter peptide presentation on MHC I molecules, thus supporting immune evasion [75,76]. The loss of β2-microglobulin is associated with acquired resistance consecutive to the initial therapeutic response to checkpoint inhibitors [54,76].

Another acquired resistance mechanism is achieved by the disruption of the IFN-γ–JAK/STAT pathway. Melanoma cells can disrupt IFN-γ signaling pathways by altering JAK1/JAK2 tyrosine kinases, which suppress interferon-mediated upregulation, immune-mediated anti-tumor defense and support resistance to immunotherapy [54,77].

TME endorses immune evasion by creating an immunosuppressive setting which consists of Tregs, TAMs and MDSCs, which impair effector T-cell function and augment tumor progression [78]. TME is implicated in both primary and acquired resistance by inhibitory cytokines production, such as TGF-β, IL-10 and vascular endothelial growth factor (VEGF), which suppress T-cell activation and supports immune tolerance [78].

Furthermore, tumor cells can yield to T-cell exhaustion through sustained antigen exposure and stimulation of several inhibitory pathways. This mechanism is accomplished by the upregulation of various inhibitory receptors, such as PD-1, LAG-3 or TIM-3, which leads to both progressive impairment of effector T-cell function and enhancement of dysfunctional T-cell [79].

Moreover, a metabolic competition within the TME is created, particularly by nutrient drain concurrent with the accumulation of immunosuppressive metabolites, thus additionally interfering with T-cell function and facilitating immune evasion [80]. The most important metabolic processes include tryptophan depletion by indoleamine 2,3-dioxygenase, adenosine accumulation, and acidosis via lactate buildup, all being metabolic elements contributing to T-cell dysfunction and resistance to immunotherapy [81,82]. In some cases, melanomas exhibit poor T-cell infiltration, a mechanism associated with primary resistance, performed by the activation of oncogenic pathways such as WNT/β-catenin signaling, which impairs the immune response [83].

These resistance mechanisms altogether create a complex immune evasion strategy that enables melanoma cells to avoid immune monitoring and reduce the efficacy of anticancer immunotherapy.

## 3. Comparative Assessment of Cancer Pathways Between Malignant Melanoma and Other Types of Cancers

Melanoma, non-small cell lung cancer (NSCLC), and renal cell carcinoma (RCC) have common immunological and molecular mechanisms that support both their response and resistance to current immunotherapy. These types of tumors feature different degrees of immunogenicity, combined with elevated neoantigen burden, all of which enable T-cell recognition while promoting immune selection and evasion [84]. The central shared immune evasion mechanism consists of the activation of inhibitory immune checkpoints (PD-1/PD-L1 and CTLA-4), leading to T-cell exhaustion and impaired effector function [85]. In addition, defects in antigen presentation, including downregulation of MHC I molecules and loss of β2-microglobulin, are observed across these cancers and result in reduced tumor visibility to CTL [54]. Resistance to immune checkpoint blockade is further mediated by disruption of IFN-γ signaling pathways, frequently through alterations in JAK1/JAK2, which impair antigen presentation and immune-mediated tumor killing [54].

The TME in melanoma, NSCLC, and RCC is commonly immunosuppressive, characterized by the accumulation of regulatory T cells, myeloid-derived suppressor cells, and tumor-associated macrophages, as well as the secretion of inhibitory cytokines such as TGF-β, IL-10, and VEGF, all of which suppress T-cell activity and promote tumor progression [86]. Continuous antigen exposure linked to these cancer types induces T-cell exhaustion, exacerbated by the presence of various inhibitory receptors, including PD-1, LAG-3, TIM-3, and TIGIT, which facilitates the functional impairment of tumor-infiltrating lymphocytes [87]. Several metabolic processes within the TME, like increased glycolysis, tryptophan depletion and lactate and adenosine buildup, all contribute to an inappropriate environment for effector T cells, which further impairs antitumor immunity [88]. In addition, tumor-intrinsic pathways, such as WNT/β-catenin signaling and abnormal angiogenesis, contribute to T-cell exclusion and the development of “cold” tumors, mechanisms of primary resistance to immunotherapy [89].

The common mechanisms in these three types of cancers highlight a common foundation for developing immune evasion and resistance, providing biological justification for the implementation of immune checkpoint inhibitors therapy in these types of malignancies.

**Figure 4:**
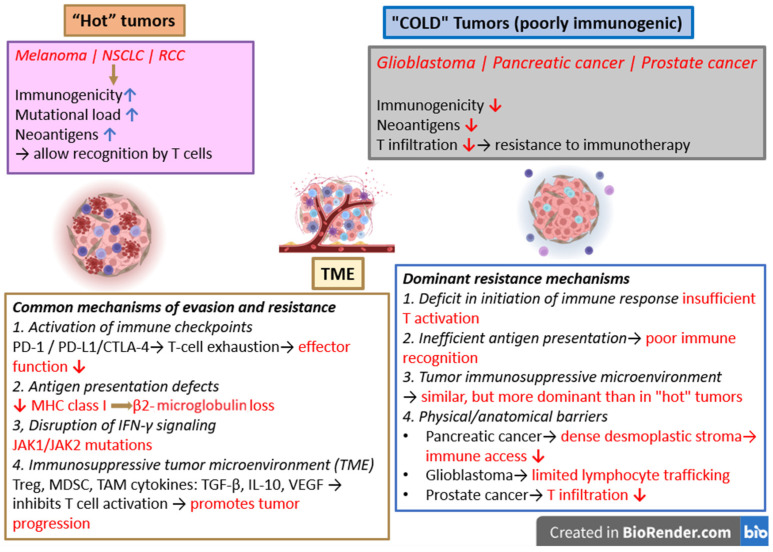
Comparative evaluation of immune evasion and immunotherapy resistance mechanisms in different types of cancer. ↓—decrease/downregulation; ↑—increase/upregulation; Other arrows—leads to/generates (Created in BioRender. Oana Bătrîna. (2026) https://BioRender.com/n3f9017).

Melanoma fundamentally differs from glioblastoma, pancreatic cancer, and prostate cancer regarding its immunological landscape and responsiveness to immunotherapy. Melanoma is classified as an immunologically “hot” tumor, characterized by a high tumor mutational burden and an elevated neoantigen load, which facilitate T-cell recognition and infiltration, although this is mitigated by immune checkpoint-mediated suppression [84,90]. Glioblastoma, pancreatic cancer, and prostate cancer are “cold” tumors, defined by low immunogenicity, limited neoantigen-mediated immune activation, and impaired infiltration of effector T cells, leading to resistance to immune checkpoint blockade [90,91] (Figure 4). Immune evasion in melanoma is predominantly facilitated by inhibitory immune checkpoints, resulting in T-cell exhaustion [84]. In contrast, resistance in glioblastoma, pancreatic cancer, and prostate cancer is primarily facilitated by mechanisms that inhibit effective T-cell priming and infiltration, including immunosuppressive tumor microenvironments, inadequate antigen presentation, and physical or anatomical barriers. Pancreatic cancer has a dense desmoplastic stroma that impairs immune-cell access to the tumor. Glioblastoma has immune privilege and limited lymphocyte trafficking in the central nervous system, and prostate cancer has low baseline immune activation and less T-cell infiltration [92,93,94]. Melanoma is a tumor with a pre-existing but suppressed antitumor immune response. On the other hand, glioblastoma, pancreatic, and prostate cancers have an absent or ineffective immune response.

## 4. Plant-Derived Compounds: Classification, Sources, and Mechanisms of Action as Potential Immunotherapy Sensitizers

### 4.1. Flavonoids

#### 4.1.1. Quercetin

Quercetin is a natural flavonol with polyphenolic structure that can be found in fruits, vegetables and medicinal plants. Its molecular structure is built on a 3,5,7,3′,4′-pentahydroxyflavone backbone and contains five hydroxyl groups that provide its antioxidant properties manifested through free radical scavenging and the ability to chelate transitional metals. Quercetin can be found in onions, apples, berries, broccoli and other dietary sources [95]. In addition, quercetin exhibits a wide plethora of biological effects, including anti-inflammatory, antiviral, antimicrobial, antiaging and anticancer [96]; as an anticancer agent, quercetin was reported to inhibit tumor proliferation in numerous types of cancer by inducing apoptosis and altering cell-cycle progression.

Quercetin was widely investigated in terms of its anticancer and immunomodulatory properties that are mainly mediated through the modulation of essential oncogenic signaling pathways such as PI3K/Akt/mTOR, signal transducer and activator of transcription 3 (STAT3), MAPK, and nuclear factor kappa-light-chain-enhancer of activated B cells (NF-κB); additionally, quercetin is able to regulate tumor suppressor pathways such as p53 and cellular redox homeostasis [97,98,99]. Subsequently, quercetin suppresses tumor proliferation, induces apoptotic processes due to the regulation of Bcl-2 family proteins and caspases (downregulation of anti-apoptotic proteins Bcl-2 and Bcl-xL, and upregulation of pro-apoptotic mediators Bax and caspases), alters intracellular reactive oxygen species (ROS) levels, and promotes cell-cycle arrest [98,100]. Moreover, quercetin is able to alter the TME and immune response in several cancer types, including melanoma. Specifically, in melanoma cells, the regulation of certain signaling pathways closely associated with immune evasion such as STAT3, PI3K/Akt, and MAPK was correlated with an increased tumor immunogenicity [101]. Due to its inhibitory effect on NF-κB signaling and the production of pro-inflammatory cytokines, quercetin alters immune cell infiltration, thus contributing to the reprogramming of the TME [100]. These findings suggest that quercetin is able to indirectly exert immunomodulatory effects by both reducing inflammation and partially reversing the immune evasion mechanisms. However, these effects can be regarded more as the result of tumor–immune interactions than as direct evidence of immune checkpoint sensitization.

In addition to acting as an immunomodulating agent, quercetin was also identified as a chemosensitizing agent. Its ability to inhibit ATP-binding cassette (ABC) transporters, particularly P-glycoprotein, triggers an enhanced intracellular drug accumulation and also improves the efficacy of associated conventional anticancer drugs (such as 5-fluorouracil, cisplatin, doxorubicin, and paclitaxel) in several cancers [102]. Nevertheless, these findings were mainly reported in non-melanoma cancers (gastrointestinal, breast, lung, prostate, hematological cancers) and cannot, therefore, be directly extrapolated to immune checkpoint blockade in melanoma. To date, preclinical or clinical data regarding the combination of quercetin with immune checkpoint inhibitors in melanoma remain scarce. Therefore, although quercetin revealed a largely recognized ability to increase tumor sensitivity to cytotoxic and immune-mediated mechanisms, the current evidence does not support its classification as a validated immunotherapy sensitizer in melanoma.

**Melanoma-specific mechanisms**

**TRAIL sensitization and apoptosis**

In melanoma cells, quercetin was revealed to increase sensitivity to recombinant human tumor necrosis factor-related apoptosis-inducing ligand (TRAIL). In MeWo and WM164 melanoma cell lines, when applied in 25–50 µM, quercetin increased the expression of the death receptor (DR)4 and DR5 receptors, promoted the activation of caspase-8, and induced the proteasome-mediated degradation of the anti-apoptotic protein cellular FLICE-like inhibitory protein (FLIP), thus inducing a dose-dependent apoptosis and partially overcoming TRAIL resistance [103]. However, it must be emphasized that this mechanism can be regarded as apoptosis signaling modulation rather than immune checkpoint sensitization and therefore cannot be directly extrapolated to PD-1/PD-L1-mediated immunotherapy. Furthermore, the concentrations used in vitro (25–50 µM) substantially exceed physiologically achievable plasma levels in humans, which are typically in the sub-micromolar range [104,105], raising concerns regarding the translational relevance of these findings.

**STAT3 signaling and tumor progression**

The important role played by STAT3 inhibition in the anti-melanoma activity exerted by quercetin was reported by Cao et al., who revealed that quercetin is able to suppress both the constitutive and inducible STAT3 phosphorylation, to reduce nuclear translocation, and to decrease the transcriptional activity [98]. This inhibitory activity leads to the downregulation of downstream STAT3-dependent genes which are involved in tumor progression (Bcl-2, Bcl-xL, Mcl-1, survivin, cyclin D1, VEGF), to an increased sensitivity to pro-apoptotic factors, and to the activation of caspase-3 and PARP cleavage.

In addition, STAT3 inhibition triggers the reduction of certain immunosuppressive and pro-angiogenic signaling pathways, thus suggesting a potential alteration of the TME and an increased tumor response to immunologic mechanisms.

While such findings are indicators of quercetin targeting signaling pathways closely related to tumor immune evasion, including STAT3-dependent immunosuppression, current evidence unfortunately remains limited to preclinical experiments. Of note, studies that directly assess its combination with immune checkpoint inhibitors against melanoma are still lacking. Therefore, although STAT3 can be regarded as a mechanistically relevant target in immuno-oncology, the potential of quercetin to function as an immunotherapy sensitizer has yet to be established.

**CD47 axis and innate immune modulation**

Quercetin was also reported to interfere with tumor immune evasion by modulating the phosphoinositide-dependent protein kinase-1 (PDK1)/CD47 axis. Thus, quercetin is able to suppress PDK1 and its downstream PI3K/Akt signaling, subsequently reducing CD47 expression at both transcriptional and protein levels, finally resulting in the stimulation of macrophage-mediated phagocytosis of melanoma cells [106]. Given that CD47 functions as a key “don’t-eat-me” signal through its interaction with signal regulatory protein alpha (SIRPα) at the macrophage level, its downregulation is able to facilitate tumor recognition by the immune system followed by tumor cell death. In vivo, these effects cause the reduction in tumor growth in melanoma-bearing mice that also showed a decreased CD47 expression, an increased macrophage infiltration, and an increased activation of immune processes; of note, these mechanisms occur while normal tissues remain unaffected.

In terms of translation from preclinical level to clinic, the targeting of the CD47–SIRPα axis stands as a relevant strategy that is currently under investigation in immuno-oncology. Thus, the suppression of CD47 by quercetin emphasized its potential role in regulating immune checkpoints, in particular against tumors with impaired antigen expression. However, this mechanism is mainly centered on macrophage-dependent immune responses instead of directly addressing immune checkpoint pathways, such as PD-1/PD-L1 or CTLA-4 signaling, which are essential in melanoma immunotherapy. Consequently, although quercetin is indeed a valuable agent in the regulation of innate immune mechanisms, its ability to function as a clinically relevant immunotherapy sensitizer still remains to be established.

**RIG-I/type I interferon signaling**

Quercetin can further modulate the innate immune signaling through the activation of the RIG-I/type I interferon pathway. More specifically, quercetin increases retinoic acid-inducible gene I (RIG-I) expression, stimulates interferon regulatory factor (IRF)3 phosphorylation, increases IFN-α and IFN-β production, and upregulates interferon-stimulated genes. Collectively, these mechanisms result in antiproliferative and pro-apoptotic effects in both murine (B16F10) and human (A375) melanoma cells [107]. The study also tested quercetin in vivo where it suppressed tumor growth in melanoma-bearing mice, accompanied by the increased expression of RIG-I and type I interferon-related genes in tumor cells, as well as an increased immune activation within the TME.

From a translational perspective, the activation of type I interferon signaling is a key factor in tumor response to the immune checkpoint blockade, since it stimulates antigen expression, the activation of dendritic cells, and, subsequently, the activation of naïve T cells. However, the efficacy of this pathway is strongly dependent on the existence of intact downstream signaling, particularly through JAK1/2-mediated interferon responses. Mutations in JAK1/2 with loss of function that are frequently described in melanoma can block interferon signaling and thus provide resistance to the PD-1/PD-L1 blockade.

Therefore, although the activation of the RIG-I/IFN-I axis by quercetin strongly supports its potential to optimize tumor immunogenicity, its clinical efficacy may be quite limited in tumors displaying defects in the interferon-signaling pathways. Moreover, direct evidence in terms of quercetin actually restoring or bypassing JAK1/2-dependent resistance mechanisms, or sensitizing melanoma to immune checkpoint inhibitors, are still missing in the literature.

**Immune checkpoint relevance**

Quercetin has been reported to modulate immune checkpoint-related pathways, including the downregulation of PD-L1 expression via the inhibition of STAT3 signaling and the potential disruption of the PD-1/PD-L1 interaction [98,108]. These effects have been associated with an increased T-cell activity as well as an increased production of effector molecules such as interferon-γ and granzyme B. Simultaneously, STAT3 inhibition hinders the transcription of tumor-promoting and immunosuppressive genes, including Bcl-2, survivin, cyclin D1, and VEGF, thus stimulating tumor susceptibility to immune-mediated cell death.

These findings suggest that quercetin is able to modulate signaling pathways that play important roles in the adaptive immune checkpoint regulation. However, this mechanistic hypothesis has not yet been translated into validated therapeutic options. To date, there are no published studies to validate synergistic effects between quercetin and clinically approved immune checkpoint inhibitors (e.g., anti-PD-1, anti-PD-L1, or anti-CTLA-4 therapies) in melanoma models.

Moreover, the clinically established resistance mechanisms to the checkpoint blockade in melanoma, such as JAK1/2 mutations and β2-microglobulin loss, which are able to alter the interferon signaling and antigen presentation [54], were not yet revealed to be directly affected by quercetin. Given that the restoration of these pathways is a key player in effective immunotherapy sensitization, this lack of evidence stands as a critical gap between mechanistic findings and clinical relevance. Collectively, although quercetin actually exhibits the ability to modulate the PD-1/PD-L1 axis and the STAT3-dependent immunosuppression, its ability to function clinically as an effective immunotherapy sensitizer in melanoma has yet to be validated.

**Multimodal strategies: photothermal therapy**

Quercetin has been investigated in addition to photothermal therapy by using nanoparticle-based delivery systems designed to optimize the overall therapeutic efficacy. Thus, its entrapment into iron-based nanoparticles facilitates the photothermal conversion under near-infrared irradiation, finally resulting in increased tumor cell death and the modulation of the immunosuppressive TME [109]. This combination was associated with the alteration of macrophage phenotypes, with reduced immunosuppressive M2 macrophages and increased levels of pro-inflammatory M1 macrophages that indicate lower immune suppression and stronger antitumor immune activity. Moreover, the combined treatment triggered signs of immunogenic cell death that included increased calreticulin exposure and the release of damage-associated molecular patterns (DAMPs) such as high-mobility group box 1 protein (HMGB1) and ATP, which subsequently stimulate the activation of dendritic cells followed by T-cell priming.

**Figure 5:**
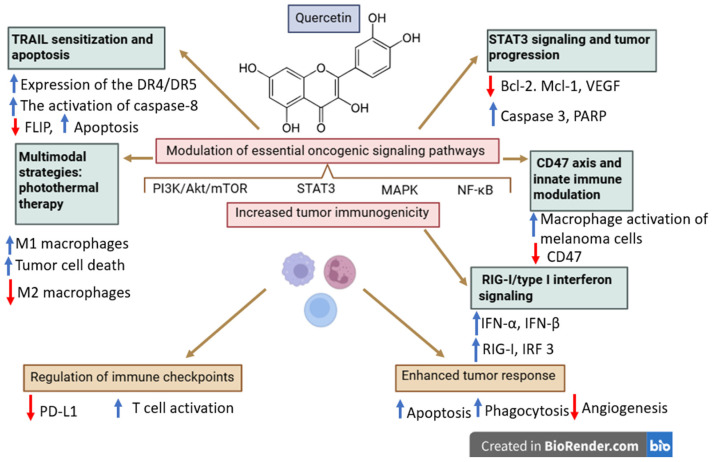
The specific mechanism of quercetin in melanoma includes TRAIL sensitization and apoptosis, STAT3 signaling and tumor progression, CD47 axis and innate immune modulation, RIG-I/type I interferon signaling, and photothermal therapy. ↓—decrease/downregulation; ↑—increase/upregulation; Other arrows—leads to/generates (Created in BioRender. Oana Bătrîna. (2026) https://BioRender.com/csn8cr0).

The study also reported a reduction in the PD-L1 expression due to the inhibition of the JAK2/STAT3 signaling pathway (Figure 5). However, this effect occurred within a complex nanoparticle-based photothermal system and should therefore be interpreted in the context of multimodal therapy rather than as direct evidence of immune checkpoint blockade or sensitization to anti–PD-1/PD-L1 therapies. In the same note, the increased cytotoxic T-cell responses are more likely determined by the increased antigen release and presentation as a result of immunogenic cell death than by the specific modulation of the checkpoint signaling. In terms of preclinic-to-clinic translation, these findings emphasize quercetin’s potential to act as an anticancer agent within a multifunctional therapeutic platform rather than as a standalone immunotherapy sensitizer. However, one cannot isolate its individual contribution to the immune checkpoint modulation and thus its relevance in clinically approved immunotherapies requires further validation.

**Resistance mechanism alignment**

The resistance to the immune checkpoint blockade in melanoma is frequently caused by defects in interferon signaling (e.g., JAK1/2 mutations) and antigen presentation (e.g., β2-microglobulin loss), which hinder tumor recognition and clearance by cytotoxic T cells [54,110]. In terms of preclinic-to-clinic translation, the restoration of these pathways stands as a key factor in achieving an effective immunotherapy sensitization. Unfortunately, to date, there are no studies to clearly demonstrate quercetin’s ability to reverse these resistance mechanisms. Consequently, this limitation can be considered the most difficult to overcome barrier in its development as a clinically relevant immunotherapy sensitizer.

**Pharmacokinetic limitations**

Despite its highly promising biological activity, quercetin exhibits unfavorable pharmacokinetic properties, specifically low aqueous solubility, fast first-pass metabolism mainly through glucuronidation and sulfation, rapid systemic clearance, and extremely low oral bioavailability (<1%). Consequently, its plasma concentrations in humans usually remain within the sub-micromolar range, far below those able to exert effective in vitro anticancer activity [111,112,113]. This pharmacokinetic profile is a critical gap between preclinical activity and clinical applicability and thus represents a major barrier for quercetin development as a therapeutic agent.

**Delivery strategies and translational implications**

Quercetin has been widely investigated in order to optimize both its pharmacokinetic profile and its tumor-targeting ability, resulting in the development of advanced delivery systems such as polymeric nanoparticles, liposomes, micelles, and lipid-based nanocarriers [114,115,116]. These delivery systems are designed to overcome quercetin’s intrinsic limitations related to aqueous solubility, stability, and rapid metabolism, thus increasing systemic exposure and prolonging its circulation time [114], as well as promoting tumor accumulation through the enhanced permeability and retention (EPR) effect [115].

Polymeric nanoparticles, usually containing biodegradable polymers such as poly(lactic-co-glycolic acid) or chitosan, are able to improve quercetin stability and ensure its protection against enzymatic degradation while also enabling its controlled and sustained release [117]. In addition, a huge advantage is that their surface can be functionalized with targeting ligands, thus facilitating receptor-mediated cellular uptake and increasing tumor specificity [118].

Liposomes, composed of phospholipid bilayers, allow the encapsulation of hydrophobic compounds such as quercetin within their lipid membrane, thus improving their aqueous dispersion and providing their protection against oxidative degradation [116]. PEGylated liposomes further reduce quercetin’s opsonization and clearance by the reticuloendothelial system, thus prolonging its circulation time and increasing its tumor accumulation [114].

Polymeric micelles, consisting of amphiphilic block copolymers, are able to solubilize quercetin within their hydrophobic core while maintaining its stability in aqueous media, thus enabling an improved tumor penetration due to their nanoscale diameters [115]. Such systems can also be engineered to enable drug release as a result of external stimuli within the tumor-specific environment (e.g., acidic pH, redox gradients) [114].

Lipid-based nanocarriers, including solid lipid nanoparticles and nanostructured lipid carriers, are able to increase drug solubilization and facilitate its lymphatic uptake, thus partially bypassing the first-pass metabolism and improving quercetin’s oral bioavailability [116]. These systems have also revealed an improved skin penetration and local drug delivery, which is particularly useful in the treatment of cutaneous melanoma [119]. In agreement with these mechanistic advantages, such nanoformulations have also been shown to improve tumor retention and result in an increased overall therapeutic efficacy in melanoma models [120].

However, despite these significant improvements in pharmacokinetic parameters and tumor delivery, it remains unclear whether such systems can actually achieve tumor concentrations high enough to modulate the immune checkpoint pathways in vivo. From a translational perspective, this emphasizes a critical limitation: an increased delivery does not necessarily translate into an effective immunotherapy sensitization, particularly in the absence of a direct effect on resistance-driving mechanisms.

**Linking delivery to immunotherapy**

The integration of these advanced delivery systems with immunotherapy stands as a promising strategy to overcome the well-known quercetin limitations. By increasing the systemic exposure to quercetin and its tumor-specific accumulation, nanocarriers may actually enable the achievement of high enough concentrations able to modulate immune checkpoint pathways in vivo. Moreover, such systems also allow quercetin co-delivery with immune checkpoint inhibitors, its targeted release within the TME, and its combination with other immunomodulatory agents. These advantages are particularly useful in melanoma, where an effective immunotherapy strongly depends on achieving adequate tumor drug levels and the activation of immune mechanisms. However, there is currently no direct evidence to support the fact that quercetin, even when delivered via advanced nanocarriers, is able to increase the efficacy of immune checkpoint blockade in melanoma. This contrasts with other phytochemicals, such as genistein, for which synergistic effects with anti-PD-1 therapy have already been reported [121].

**Chemical derivatization strategies**

Another direction to overcome the well-documented pharmacokinetic limitations of quercetin consists of its chemical derivatization in order to increase absorption, metabolic stability, and membrane permeability; of note, chemical derivatives were mainly synthesized by involving the numerous hydroxyl groups grafted on the main scaffold.

The most consistently validated approach is through glycosylation, particularly the synthesis of quercetin glucosides. Pharmacokinetic studies conducted in humans have revealed that quercetin-3-O-glucoside and quercetin-4′-O-glucoside are absorbed more efficiently than their respective aglycones, due to their active transport via intestinal glucose transporters [122,123]. Further optimization through its enzymatic α-oligoglucosylation, as in the enzymatically modified isoquercitrin, has been reported to significantly optimize oral bioavailability as well as systemic exposure, thus standing as one of the most robustly validated derivatization strategies in humans [124].

Another widely explored strategy in quercetin’s chemical modulation is its O-methylation which reduces its tendency to sustain rapid phase II metabolism by hiding its reactive hydroxyl groups. Methylated derivatives such as isorhamnetin were shown to exhibit an increased metabolic stability and prolonged systemic circulation, although direct evidence of improved human bioavailability remains more limited compared to glycosylated species [125,126].

Two additional derivatization approaches include quercetin’s esterification and acylation, which increase its lipophilicity and thus enable its passive membrane diffusion. Regioselective acylated derivatives have exhibited improved physicochemical parameters (e.g., solubility, stability) and an optimized biological activity in preclinical studies, collectively supporting their potential to modulate quercetin pharmacokinetics [127,128]. Furthermore, metal–quercetin complexes [129,130] and other semisynthetic analogs [131] have been investigated to improve chemical stability and intracellular accumulation, although these strategies remain less well characterized from a translational pharmacokinetic perspective.

From a translational perspective, both the preparation of advanced delivery systems and the synthesis of chemical derivatives aim to increase systemic exposure; however, chemical derivatization aims to generate analogs with intrinsic improved properties while nanoformulations involve the dependency on external carriers. Of note, neither strategy has yet demonstrated the ability to provide the consistent modulation of immune checkpoints or to overcome key resistance mechanisms in melanoma, thus indicating the persistent gap between pharmacokinetic optimization and clinically relevant immunotherapy sensitization.

This limitation suggests that, even when bioavailability is substantially improved, quercetin and its derivatives may remain limited in terms of clinical efficiency by insufficient target specificity toward resistance-related pathways, such as interferon signaling defects and antigen presentation loss; therefore, one may state that pharmacokinetic optimization alone is likely insufficient to enable the effective immunotherapy sensitization in melanoma. Collectively, quercetin can be more appropriately regarded as a potential adjuvant or component of combined strategies rather than a standalone immunotherapy sensitizer.

#### 4.1.2. Luteolin

Luteolin is a natural flavonoid containing the 3′,4′,5,7-tetrahydroxyflavone ring; it can be extracted from various medicinal and edible plants such as celery, parsley, green pepper, chamomile and thyme. Due to the presence of several hydroxyl groups in its structure, luteolin is able to act as an antioxidant and also displays anti-inflammatory and anticancer effects. Similarly to quercetin, luteolin is able to modulate several pathways revealed to be involved in tumor progression as well as tumor immune evasion, such as STAT3, PI3K/Akt, MAPK, and NF-κB signaling pathways. Despite its well-known antitumor and immunomodulatory activity, the reported melanoma-specific evidence is still limited and should be therefore interpreted separately from the data available in non-melanoma cancers. Specifically, the vast majority of literature data regarding luteolin used as a sensitizer focus on other cancer types and thus cannot be directly extrapolated to the immune checkpoint blockade in melanoma.

**Melanoma-specific mechanisms**

**STAT3 signaling, survival, and invasion**

Li et al. reported luteolin’s direct anti-melanoma effects in several types of melanoma cells as well as in in vivo mice models obtained by means of murine B16F10 and human A375 xenografts. Luteolin achieved a reduced cell viability in vitro where it induced apoptosis and inhibited cell migration [132]. In terms of underlying mechanisms, luteolin was found to inhibit Src-dependent STAT3 phosphorylation, thus enabling STAT3 ubiquitination and proteasomal degradation, finally leading to the suppression of downstream STAT3-regulated genes that act as key players in tumor survival and invasion. Since STAT3 can be regarded as an important regulation factor in melanoma progression and contributes essentially to tumor-related immunosuppression, one can state that such findings are highly relevant in immuno-oncology. However, they should be interpreted more like evidence for pathway modulation than a direct proof of immunotherapy sensitization.

Additional studies were reported that support this anti-survival effect; in A375 melanoma cells, luteolin was reported to inhibit the PI3K/Akt-associated signaling pathway and also reduced the matrix metalloproteinase (MMP)-2/MMP-9 expression, thus exerting a significant anti-metastatic effect [133]. In another work [134], the authors reported that luteolin induced changes in melanoma cells following three main aspects: the cell–cell interacting pathway (extracellular matrix), the oncogenic pathway, and the immune response signaling pathway. Earlier data revealed the suppression of the β3-integrin-associated cell invasion and epithelial–mesenchymal transition-like behavior in melanoma cells [135]. Collectively, these findings emphasize the fact that luteolin is able to interfere with melanoma cell survival, migration and metastatic ability, but data regarding its potential synergy with the already approved immune checkpoint inhibitors is still missing.

**Immune-oriented evidence in melanoma**

Important data regarding luteolin’s immune-oriented anti-melanoma activity was published by Tian et al. who showed that the phytochemical acted as an adjuvant in a B16F10 mouse model where it increased APC activation, IL-12-associated signaling, CTL responses and overall tumor control [136]. Luteolin was able to stimulate the antitumor immune activation and improve the efficacy of an anti-melanoma vaccine in vitro and in vivo; thus, it can presumably modulate the adaptive antitumor immunity. However, it must be emphasized that in this case, luteolin acted only as a vaccine adjuvant and not specifically on the immune checkpoint blockade. Therefore, although the study reported the immunomodulatory role of luteolin in melanoma cells, it did not provide evidence on the potential of the compound to reverse the resistance to anti-PD-1, anti-PD-L1, or anti-CTLA-4 therapies.

Currently, there are no studies published specifically on melanoma where luteolin might restore immune sensitivity in tumors bearing clinically relevant resistance mutations such as JAK1/2 pathway mutations, β2-microglobulin loss, or other forms of altered antigen presentation [54]. This stands as a significant translational gap since such resistance mechanisms are the main cause of failure of the immune checkpoint blockade in melanoma. Consequently, one might state that, based on the current literature, luteolin may be regarded as an immunologically active molecule, at least at the preclinical level, but cannot yet be considered an immunotherapy sensitizer in melanoma.

**Chemosensitization vs. checkpoint sensitization**

The most direct evidence for luteolin as an anti-melanoma agent in combination therapies currently concerns chemotherapy instead of immunotherapy. Gahlot and Kang reported that luteolin was able to increase the cytotoxic effect of 5-fluorouracil in B16F10 melanoma cells by stimulating ROS production, mitochondrial dysfunction, caspase-dependent apoptosis, and cell-cycle arrest in G1-phase [137]. These findings indicate its potential to act as a chemosensitizer in vitro but do not bear any relevance to the immune checkpoint blockade. Moreover, in a similar manner to other phytochemicals, unless the concentrations necessary for the synergistic effects can be achieved in vivo, their translational significance is rather limited. Thus, the current evidence in melanoma studies is more significant for apoptosis-based or chemotherapy sensitization than for actual immunotherapy sensitization.

**Cross-tumor immunotherapy relevance**

**Figure 6:**
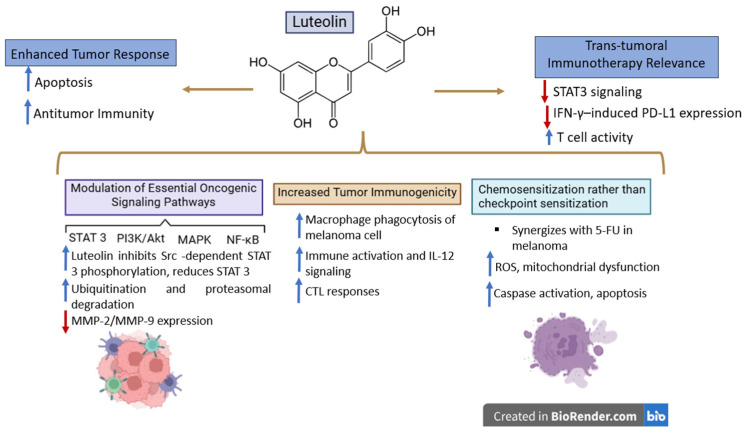
The specific mechanism of luteolin in melanoma includes modulation of essential oncogenic signaling pathways, increased tumor immunogenicity, chemosensitization rather than checkpoint sensitization, and trans-tumoral immunotherapy relevance. ↓—decrease/downregulation; ↑—increase/upregulation; Other arrows→ leads to/generates (Created in BioRender. Oana Bătrîna. (2026) https://BioRender.com/t2g6lcq).

Due to the fact that melanoma and non-small cell lung cancer are both immunotherapy-responsive tumors in which the adaptive immune resistance and the regulation of the PD-1/PD-L1 axis play key roles, data reported in NSCLC may still provide some mechanistically relevant details. Jiang et al. reported that, in KRAS-mutant NSCLC cells, luteolin caused the downregulation of the IFN-γ-induced PD-L1 expression by inhibiting STAT3 and enhancing anticancer efficacy when administered in combination with the PD-1 blockade [138] (Figure 6). These findings might provide relevance in melanoma where the inducible PD-L1 expression and the IFN-γ-associated immune adaptation are the major players in determining tumor response. However, the NSCLC result remains confined to the specificity of lung cancer and cannot be used as validation for melanoma. It should be, therefore, regarded merely as a mechanistic support for melanoma-specific studies and not as clear evidence that it might function as an immune checkpoint sensitizer in melanoma.

**Resistance mechanism alignment**

Considering the possibility of preclinic-to-clinic translation, an important aspect is whether luteolin may actually alter the resistance mechanisms directly involved in the failure of the immune checkpoint therapy in melanoma. Currently, the answer to that particular issue remains only partially answered since, although luteolin is able to modulate STAT3 and increase the CTL-associated responses in preclinical models, it still lacks direct evidence in terms of its potential to either restore immune signaling in tumors bearing JAK1/2 loss-of-function mutations or lacking β2-microglobulin or bypass those pathways through alternative immune activation pathways.

At present, the answer remains incomplete. Although luteolin modulates STAT3 and can enhance CTL-associated responses in preclinical models, there is still no direct evidence that it restores signaling in tumors with JAK1/2 loss-of-function defects, rescues antigen presentation in β2-microglobulin-deficient tumors, or bypasses those pathways through alternative immune activation routes [54]. Therefore, the current data allows the hypothesis that luteolin might play a key role in melanoma immunotherapy sensitization but does not yet support it as a validated agent in overcoming resistance. This difference is highly relevant since the modulation of immune-related pathways alone may not necessarily ensure the reversal of clinically relevant checkpoint resistance.

**Pharmacokinetic limitations**

Although luteolin exhibits highly promising biological effects, it is also well known for its pharmacokinetic flaws that hinder its translational potential, such as poor aqueous solubility, extensive phase II metabolism and poor systemic exposure of the free aglycone after oral administration. Certain pharmacokinetic studies in rats revealed that the plasma concentration of free luteolin decreases rapidly following oral administration and the conjugated metabolites of the compound can be found predominantly in the circulation. Subsequent studies conducted both on rats and humans revealed similar results with luteolin undergoing extensive metabolism with the formation of sulfate/glucuronide conjugates that are further metabolized in different ways and therefore differ in their anti-inflammatory effects [139]. Various reviews published on luteolin’s pharmacological activity reported its low bioavailability as one of the major obstacles in its translation to clinical application [140,141]. Consequently, its reported in vitro efficacy as an anticancer agent at micromolar concentrations cannot guarantee its therapeutic efficacy unless more data is available on the possibility to achieve comparable plasma levels in vivo at the tumor site.

**Delivery strategies and translational implications**

In order to address its pharmacokinetic limitations, luteolin was formulated in various nanodelivery systems aimed at improving its aqueous dispersion, protect it against premature degradation and phase II metabolism, prolong its systemic circulation, and increase exposure at the tumor site. Such advanced systems are designed to increase the fraction of bioactive luteolin able to reach the tumor tissue by reducing its crystallinity, increasing its colloidal stability and facilitating its transport through biological barriers. Additionally, nanoformulations are able to facilitate tumor accumulation through the EPR effect while simultaneously stimulating cellular uptake by endocytic mechanisms which are more effective than simple diffusion uptake. In melanoma, Fu et al. reported the preparation of luteolin-loaded nanoparticles by using the ROS-responsive poly(propylene sulfide)-poly(ethylene glycol) as a carrier in order to enhance its water solubility and accelerate its release in melanoma cells; such nanoparticles provided optimized in vitro antitumor efficacy compared to free luteolin [142]. This study is therefore providing a concrete solution to address two luteolin’s major translational flaws, poor solubility and low bioavailability. However, the increased tumor exposure cannot be directly linked to the modulation of the PD-1/PD-L1-related adaptive resistance, the restoration of antigen presentation, or the reversal of established immune-evasion mechanisms.

Luteolin was also incorporated in polymeric nanoparticles, micelles, liposomes, phospholipid complexes, and other systems with improved water solubility aimed at improving its oral absorption and systemic bioavailability [143,144,145,146,147]. Polymeric nanoparticles, mainly those containing biodegradable matrices, are able to incorporate luteolin inside a protective core, thus reducing its chemical degradation (hydrolysis, oxidation) and, subsequently, its premature metabolism, thus enabling its controlled or sustained release. Micellar systems built from amphiphilic block copolymers are particularly relevant for the formulation of poor water-soluble luteolin (and other flavonoids as well) due to the fact that they are able to solubilize the hydrophobic compound inside their lipophilic core while maintaining a hydrophilic exterior that ensures its solubilization in aqueous media. This increase further facilitates intestinal absorption and increases the apparent concentration gradient available for tissue penetration. Liposomes and phospholipid complexes incorporate luteolin into the lipid bilayers or phospholipid clusters, thus enhancing its membrane interaction, improving its stability within biological fluids and even increasing its oral/parenteral bioavailability. The additional surface modulation with PEG or various ligands may further reduce opsonization, extend the phytocompound’s circulation time and potentially improve its overall tumor selectivity.

These platforms are quite attractive from a translational perspective given the fact they address the classical gap between the flavonoids’ strong in vitro activity and weak in vivo exposure. However, most currently available nanoformulations designed for luteolin remain studied at the preclinical level only with few being assessed in melanoma models that test immunotherapy-relevant outcomes. The data is still scarce on the possibility that luteolin nanoformulations are able to achieve intratumor concentrations large enough to ensure not only a direct cytotoxic effect but also the sustained remodeling of the immune signaling pathways. This distinction is essential since a formulation may be able to improve tumor accumulation and enhance apoptosis or inhibit tumor growth but still fail to achieve the level of exposure required to modulate immune checkpoints, affect the IFN-γ-responsive signaling or regulate the TME in a manner that improves tumor response to anti-PD-1/PD-L1 therapy. Thus, one may state that delivery optimization enables superior therapeutic outcomes to free luteolin, but there is insufficient data on their ability to provide immunotherapy sensitization in melanoma. Consequently, at this point, nanoformulation has to be considered as an actual strategy to improve pharmacokinetic parameters and not as a decisive means of overcoming translational barriers.

**Chemical derivatization strategies**

Chemical derivatization has emerged as another strategy to optimize luteolin’s pharmacokinetic profile by modulating its physicochemical properties; this strategy is particularly relevant for luteolin that displays numerous phenolic hydroxyl groups that, although highly significant for its redox biological activity, strongly contributes to its low membrane permeability, high instability within physiological environments, and fast phase II metabolism (glucuronidation, sulfation). The chemical modulation aims to increase the phytocompound’s intestinal absorption, reduce its metabolic degradation or conjugation and prolong the circulation time of its bioactive form.

One direction is the development through microbial biotransformation of luteolin monophosphate derivatives that were proposed as prodrugs with optimized oral bioavailability in rats [148]. Mechanistically, the synthesis of phosphate derivatives may increase water solubility, thus facilitating gastrointestinal absorption followed by enzymatic dephosphorylation that generates the active aglycone.

Glycosylated derivatives of luteolin are also relevant given that the sugar moieties can significantly alter the intestinal transport, its solubility and metabolic fate. Depending on the monosaccharide used and its substitution site, the glycosylated derivatives may exhibit different uptake routes compared to the parent compound; however, glycosylation can also diminish its passive diffusion and alter the intracellular release of bioactive species [149,150].

Similarly, the methylation of phenolic hydroxyl groups in luteolin may lead to compounds less prone to undergoing rapid phase II conjugation, thus increasing its metabolic stability by hiding the most reactive sites on the flavonoid scaffold. This strategy is able to increase membrane permeability and prolong circulation time, but it can also modify target affinity and weaken the interactions at the tumor site that depend on the initial chemical structure [151,152]. Therefore, the pharmacokinetic advantages of methylated or other luteolin analogs must always be assessed against the possibility of reduced biological potency.

Additional semisynthetic derivatizations, including esterification [153] and other lipophilicity-increasing modulations, stand as a promising strategy to increase luteolin’s passive transmembrane diffusion and improve its incorporation into lipid-rich environments. This strategy may also lead to prodrugs that are further metabolized following their absorption or intracellular uptake, thus producing free luteolin in a controlled manner. Collectively, such chemical modulations may improve tissue distribution by regulating the lipophile–hydrophile balance. However, the translational challenge does not only consist in increasing its total systemic exposure but also in either preserving or restoring the fraction of free luteolin that is able to actively engage the relevant target at the tumor site. Thus, the chemical derivatization strategy cannot be assessed by the improved plasma levels or the oral absorption alone but also by the maintenance of the pharmacodynamic profile of the parent compound.

If comparing chemical derivatization to nanoformulation from a translational perspective, one may state that it provides an important advantage, consisting of achieving improved intrinsic pharmacokinetic behavior; nonetheless, the current evidence cannot be regarded as immuno-oncological since there is still no direct evidence that luteolin derivatives are able to modulate the PD-1/PD-L1 axis in melanoma, restore antigen presentation mutations, or overcome the resistance mechanisms related to the JAK/STAT dysfunction. Thus, the semisynthetic approach should currently be considered as a preliminary step in the development of future anti-melanoma alternative drugs and not as proof that luteolin has already become a clinically relevant immunotherapy sensitizer. Indeed, an optimized bioavailability is a necessary but not sufficient condition for translational progress unless it is accompanied by direct evidence that such derivatives are able to alter the immune pathways relevant to the response to the checkpoint blockade.

**Linking optimization to immunotherapy relevance**

In terms of preclinic-to-clinic translation, both the advanced delivery systems and the semisynthetic derivatization aim to solve the same main issue: the gap between a strong in vitro activity and an insufficient in vivo exposure. For luteolin, similar to other flavonoids, this is a milestone before its serious consideration as a useful immunotherapy agent in melanoma. However, the improvement of its pharmacokinetic profile is not sufficient due to the fact that, even if tumor exposure is increased, luteolin still has to be validated as directly acting against melanoma in combination with anti-PD-1, anti-PD-L1, or anti-CTLA-4 therapies; also, its relevance in the presence of resistance mechanisms such as interferon-signaling defects or impaired antigen presentation still has to be established.

#### 4.1.3. Apigenin

Apigenin (4′,5,7-trihydroxyflavone) is a natural flavone containing a C6-C3-C6 skeleton and three hydroxyl groups that provide its redox activity as well as its ability to interact with proteins and cell membranes [154]. It is widely distributed in both edible and medicinal plants, including parsley, oregano, celery and chamomile, therefore being a common ingredient in the human diet [155]. Apigenin was extensively investigated for its pleiotropic pharmacological activities such as antiphlogistic, antispasmodic, antibacterial agent, anti-asthmatic, and anti-Parkinson [156]. Importantly, apigenin exhibits antioxidant and anti-inflammatory effects through the scavenging of ROS and the inhibition of pro-inflammatory mediators such as COX-2 and the inducible nitric oxide synthase [157]. In terms of anticancer activity, apigenin revealed antiproliferative and pro-apoptotic effects in numerous types of cancer cells (breast, prostate, ovarian, lung, liver, pancreatic, colon, cervical) by inducing cell cycle arrest usually at the G2/M phase and also activating intrinsic apoptosis via molecular mechanisms potentially including the activation of caspase-3, -8, Bax, and TNF-α, the inhibition of Bcl-2, MMP-2, -9, Snail, and Slug, the degradation of the activated proteasomal Her2/neu protein as well as a decreased expression of NF-κB, PI3K, AKT, phospho-AKT, p38, MAPK, extracellular signal-regulated kinase (ERK), and JNK [158]. Besides direct cytotoxicity, apigenin alters tumor angiogenesis, metastasis and the immune TME, which justifies its investigation as a sensitizing anticancer agent [159].

However, in the narrow context of melanoma immunotherapy sensitization, the currently available data must be interpreted carefully; although apigenin has indeed shown a pleiotropic activity in various types of cancer, this activity cannot be directly translated to melanoma, particularly considering the specific immune checkpoint blockade where resistance is triggered by highly specific molecular defects and microenvironmental alterations.

**Melanoma-specific evidence**

A major limitation in the current literature is the fact that non-melanoma studies are frequently used to support similar claims in melanoma. Although apigenin has been indeed reported to enhance the activity of chemotherapeutic or cytotoxic agents in colon cancer, glioma, colorectal cancer, leukemia, and prostate cancer models, these studies should not be considered evidence of melanoma immunotherapy sensitization. They can, however, indicate that apigenin is able to regulate apoptosis, survival signaling pathways, and tumor response; on the other hand, they cannot validate the hypothesis that apigenin is actually able to overcome the melanoma-specific resistance mechanisms that are often responsible for the failure of anti-PD-1, anti-PD-L1, or anti-CTLA-4 therapies in clinical practice. Therefore, such cross-organ data can only be interpreted as mechanistic support and not definitive proof.

**Direct anti-melanoma activity vs. checkpoint sensitization**

**Figure 7:**
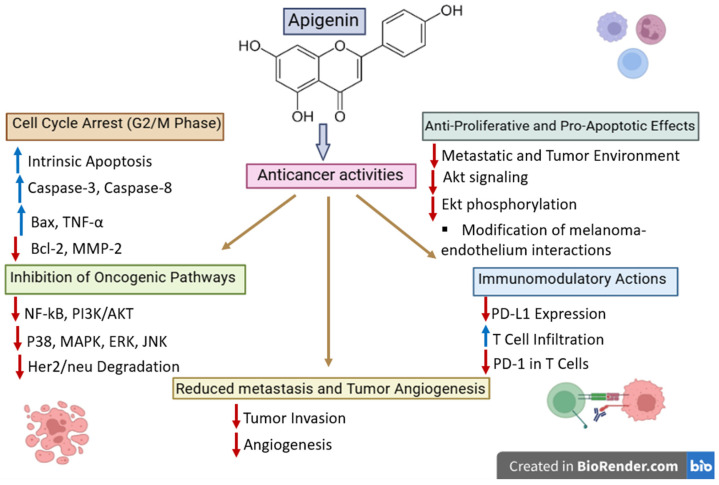
Mechanism of apigenin as a potential chemosensitizing and immunomodulatory agent in melanoma. ↓—decrease/downregulation; ↑—increase/upregulation; Other arrows—leads to/generates (Created in BioRender. Oana Bătrîna. (2026) https://BioRender.com/gd88mnd).

In the melanoma models reported in the literature, apigenin lacks direct anticancer effects in melanoma; Woo et al. reported that apigenin was able to reduce A375 melanoma cell viability by inducing apoptosis through the inhibition of Akt signaling and the regulation of MAPK pathways, which included the inhibition of ERK phosphorylation combined with the stimulation of stress-associated signaling, collectively promoting intrinsic apoptotic mechanisms [160] (Figure 7). Earlier work also revealed its anti-metastatic potential in melanoma through the inhibition of cell migration and the alteration of melanoma–endothelial interactions in a B16-BL6 melanoma model in vivo where apigenin was administered intraperitoneally [161,162]. Such studies are relevant in validating the anti-melanoma potential of apigenin due to cytotoxic and anti-metastatic effects but cannot serve as proof for its potential to restore tumor sensitivity to immune checkpoint inhibitors.

**Immune-oriented activity in melanoma**

More compelling evidence is provided by studies that revealed apigenin as an effective modulator of the PD-1/PD-L1 axis in melanoma. Xu et al. revealed that apigenin is able to downregulate the IFN-γ-induced PD-L1 expression in melanoma cells and to reduce the PD-L1 expression in dendritic cells through the inhibition of STAT1 phosphorylation, finally leading to an increased antitumor response mediated by T-cell lymphocytes [163]. Apigenin also inhibited the growth of A375 melanoma xenograft in vivo where increased T-cell infiltration was recorded in tumor tissues. The study concluded that apigenin was able to block melanoma growth via multiple mechanisms, including the suppression of the PD-L1 expression that exerted a dual effect: it regulated both tumor and antigen-presenting cells (Figure 7). This is one of the strongest pieces of evidence in terms of apigenin’s specific anti-melanoma activity because it connects apigenin more to a clinically relevant immunoregulatory pathway instead of merely an apoptotic mechanism. Nonetheless, the study still does not address the challenge of reversing resistance to approved checkpoint inhibitors in clinically resistant melanoma. It indeed shows apigenin’s ability to modulate specific immune pathways in preclinical settings, thus providing significant mechanistic evidence for its potential use as an immunotherapy sensitizer in melanoma, but direct evidence is still missing.

**Resistance mechanism alignment**

Recent work by Yang et al. showed that apigenin is able to sensitize A375, A2058, and B16F10 melanoma cells to dacarbazine and ionizing radiation while also stimulating immune cell death and reducing PD-1 expression in T cells via the proteasomal pathway [164]. The significance of this study relies on the fact that it proves that apigenin can indeed increase tumor response in melanoma when administered in combination with other therapeutic approaches; conversely, it does not provide evidence that apigenin can overcome melanoma clinical resistance to checkpoint blockade. More specifically, we were not able to find evidence that apigenin would be capable of restoring interferon sensitivity in JAK1/2-deficient melanoma, to rescue antigen presentation in tumors exhibiting β2-microglobulin loss, or to bypass such mutations through alternative immune activation pathways. This is a critical distinction given that the modulation of the PD-L1 axis or the increase in T-cell cytotoxicity at preclinical level is not necessarily associated with the reversal of clinically relevant resistance mechanisms.

Apigenin may also be assessed against other immune-responsive tumors, particularly NSCLC and RCC, because these malignancies, similar to melanoma, stand as clinically relevant models for checkpoint-based immunotherapy. Nevertheless, such comparisons can be used in a narrower sense only for mechanistic support and not as evidence that similar effects necessarily occur in melanoma.

In lung cancer, Jiang et al. reported that both luteolin and apigenin were able to suppress the inducible PD-L1 expression and thus improve antitumor immunity in KRAS-mutant lung cancer [138]. Mechanistically, this is a highly relevant aspect because the regulation of inducible PD-L1 is also an essential factor in adaptive immune resistance in melanoma. Additional NSCLC studies in A549 and H1299 cells revealed that apigenin stimulated TRAIL activity through the upregulation of DR4/DR5 receptors in a p53-dependent manner [165], inhibited the proliferation, migration, and invasion of A549 human lung cells via the PI3K/Akt pathway [166], and also enhanced the antitumor effect of cisplatin in A549, H1299, and cisplatin-resistant NSCLC A549R cells by inhibiting cancer stem cell properties [167]. Collectively, the results indicated that apigenin significantly interferes with apoptosis resistance, stem cell-associated tumor survival, and PD-L1-related immune evasion, mechanisms that are also highly relevant in melanoma. However, they still do not definitively prove that apigenin may overcome melanoma-specific checkpoint resistance.

In RCC, the available evidence shows a more indirect relation to the checkpoint signaling but can still be informative from a translational perspective. Meng et al. revealed that apigenin inhibited RCC cell proliferation by inducing DNA damage, causing cell cycle arrest at G2/M level and triggering p53-dependent apoptosis; the antitumor effect was also supported in in vivo xenograft nude mouse models [168]. 

A later study confirmed apigenin’s ability to inhibit the proliferation of human Caki-1, ACHN and NC65 RCC cells through G2/M phase cell-cycle arrest and further demonstrated that apigenin also reduces cyclin A, B1, D3, and E expression levels [169]. Of note, RCC also provides a more concrete example of a potential chemical optimization, since a newly developed apigenin derivative was reported to suppress RCC by directly inhibiting wild-type and mutant MET, the receptor of hepatocyte growth factor, and a key player in the development and progression of RCC; it also is a validated drug target in RCC treatment [170]. Nonetheless, even the activity of this derivative can at best be interpreted as proof of developmental strategies and not as the validation needed by apigenin-derived scaffolds as successful agents in overcoming the immune checkpoint resistance in melanoma.

Within the scope of this review, the main translational challenge is not whether apigenin is able to alter specific survival pathways or even the PD-L1 expression but whether it is able to reverse the specific resistance mechanisms involved in the failure of checkpoint blockade in patients. The current data can only provide an incomplete answer because, although apigenin clearly modulates the Akt, MAPK, STAT1 and PD-L1-associated signaling in melanoma cells, there are no studies to validate its ability to restore tumor sensitivity in tumors bearing loss-of-function mutations that modulate the interferon signaling, antigen presentation or the immune evasion phenotypes. Therefore, apigenin can be regarded as a preclinical candidate as an immunotherapy sensitizer due to its mechanistic overlap with some significant immune pathways.

**Pharmacokinetic limitations**

An important challenge is the gap usually reported between the significant in vitro activity and the achievable systemic exposure; similar to many compounds in its class, apigenin exhibits poor water solubility and oral absorption as well as an extensive phase II metabolism that are responsible for its low bioavailability. Consequently, the micromolar concentrations used in vitro can rarely be achieved in vivo, either in plasma or at tumor site, following conventional administration. Therefore, the mechanisms identified in vitro bear clinical significance only if evidence exists that a comparable free apigenin exposure can be achieved and maintained in vivo; otherwise, the in vitro sensitization data remains only preliminary.

**Delivery optimization and semisynthetic modification**

Given its pharmacokinetic flaws, apigenin was formulated as nanostructured systems in order to improve its pharmacological performance. One early work focused on the preparation of self-microemulsifying drug delivery systems that were able to increase its water dissolution and intestinal delivery. This approach was later developed in the preparation of self-nanoemulsifying drug delivery systems with increased transcellular absorption and oral bioavailability in vivo [171,172].

A second major strategy aimed at the reduction in particle size by solid dispersion engineering. Apigenin nanocrystals were prepared by means of a supercritical antisolvent process in order to increase its dissolution properties and subsequent oral bioavailability [173]. Similarly, mesoporous silica nanoparticles loaded with apigenin improved the phytocompound’s solubility and dissolution profile as well as its oral bioavailability [174]. In another study, a carbon nanopowder solid dispersion was prepared with similar improved physicochemical and pharmacokinetic properties [175]. Other studies focused on more complex nanocarriers that are not only able to increase dissolution but also protect the compound against degradation and facilitate its biological transport. For example, polymeric micelles containing Pluronic P123 and Solutol HS15 were reported to significantly improve the water solubility of apigenin while also providing high encapsulation efficacy

Polymeric micelles based on Pluronic P123 and Solutol HS15 were reported to markedly improve the aqueous solubility of apigenin while maintaining high encapsulation efficiency [176]. Also, a phospholipid phytosome formulation was developed, aiming to improve apigenin’s solubility, dissolution profile and bioavailability, thus emphasizing the potential of phospholipid complexation to optimize membrane interaction and oral uptake of the compound [177]. More recently, hybrid polymeric nanoparticles containing lecithin, chitosan and tocofersolan were prepared as an oral delivery system displaying improved in vitro physicochemical and cytotoxic effects [178]. Oral nano-bilosomes were developed as vesicular carriers using bile salts as stabilizers; they exhibited optimized pharmacokinetic behavior at the preclinical level [179].

Collectively, these studies indicate apigenin as not only a bioactive flavone but also as a scaffold for various types of nanoformulations that were developed in order to mitigate its pharmacokinetic flaws; however, these advances must be regarded as preliminary steps in its development toward clinical applications since the improved exposure alone does not necessarily bear relevance in melanoma treatment. Additionally, these nanoformulations were not yet revealed to overcome the clinically relevant immune-resistance mechanisms such as the JAK1/2 dysfunction or the β2-microglobulin loss; therefore, nanoformulation should be regarded not as proof that apigenin is able to overcome its major translational gap but as a necessary translational step.

Apigenin may also serve as a scaffold for semisynthetic derivatization aimed at overcoming the free aglycone’s poor water solubility, limited membrane permeability and fast metabolism. Several strategies were followed in this regard, resulting in methylation [125,180], acylation/esterification [181], synthesis of phosphate prodrugs [182] and other semisynthetic modulations [183] that allowed the establishment of structure–activity relationships and optimization.

One straightforward approach is the O-methylation that can reduce the number of phenolic hydroxyl groups involved in apigenin’s fast metabolism, thus increasing its lipophilicity. A proper example is the synthesis of acacetin and genkwanin through the lipase-catalyzed deacetylation of apigenin 5,7-diacetate followed by methylation [184]. Such methylated analogs provide knowledge on how hindering hydroxyl groups may tune the exposure-related properties of apigenin.

Another possibility consists of apigenin glycosylation; Zhao et al. reported the synthesis of rare apigenin glycosylated derivatives with significantly increased aqueous solubility (up to 174-fold) compared to the parent compound [185]. Even though the study was not focused on anticancer research, glycosylation was indicated as a potential solution to apigenin’s formulation challenge; however, such an improvement cannot translate automatically to superior biological activity since these derivatives have to undergo enzymatic hydrolysis in order to release the active aglycone and may also follow different cellular uptake and metabolic pathways compared to the free aglycone.

Apigenin may also be submitted to acylation in order to increase its lipophilicity and potentially optimize its membrane interactions. Several fatty acid esters were synthesized in a study that aimed to increase apigenin’s lipophilicity and, subsequently, its antioxidant activity [181]. Although not oriented on melanoma, this work provided proof that esterification is indeed able to optimize the phytocompound’s physicochemical profile.

Diamantis et al. reported the design and synthesis of a novel apigenin prodrug aimed at alkaline-phosphatase-instructed self-inhibition in tackling cancer [182]. Alternatively, in a recent study, monophosphate derivatives were synthesized as alternatives with increased bioavailability [148]. Although such derivatives stand as concrete chemical modulations able to address the main delivery challenges of the phytocompound, they are yet to be tested in terms of intratumor exposure, in vitro modulation of the PD-1/PD-L1 axis, and restoration of antigen presentation or potential activity in resistant melanoma bearing JAK1/2 or β2-microglobulin mutations.

Other semisynthetic derivatives were proposed as well. Liu et al. synthesized apigenin derivatives with optimized antiproliferative activity in several types of cancer cells (lung, liver, cervical, breast) [183], while a later study in RCC identified a novel derivative able to suppress RCC by directly inhibiting wild-type and mutant MET [170]. Such studies are highly useful in providing evidence that the semisynthetic modulation is able to improve drug targeting or efficacy but still cannot determine if a modified apigenin scaffold may overcome the immune checkpoint resistance in melanoma.

Overall, the chemical derivatization of apigenin must be regarded as a necessary translational tool rather than as clear evidence that the compound will be able in the future to overcome its historical limitations. The above-mentioned strategies may indeed improve selected properties such as solubility, lipophilicity, stability or bioavailability, and therefore directly address the gap between strong in vitro activity and weak in vivo exposure. However, unless such derivatives will be tested in melanoma models with clinically relevant immune-resistance phenotypes and linked to immunologically relevant endpoints, they remain at the level of merely enabling technologies rather than clear proof that apigenin can be used as an immunotherapy sensitizer.

**Overall assessment**

Overall, one may state that apigenin is one of the most mechanistically interesting flavonoids in melanoma treatment due to the direct preclinical data that shows its ability to induce apoptosis, suppress metastatic behavior, modulate PD-L1-associated signaling, and increase tumor sensitivity to chemo-/radiotherapy in melanoma models [160,163,164]. However, there is insufficient evidence to date to classify apigenin as a validated immunotherapy sensitizer in melanoma. Its ability to overcome clinically relevant resistance factors such as JAK1/2 or β2-microglobulin loss mutations is yet to be determined; additionally, there are still pharmacokinetic challenges that limit its translation from cell culture to clinical applications in humans. Consequently, apigenin stands as a promising preclinical scaffold whose future relevance will depend on two still unmet conditions: the demonstration of its activity in melanoma models bearing checkpoint-resistance mechanisms and the development of delivery or semisynthetic strategies able to achieve the required tumor site exposure.

#### 4.1.4. Epigallocatechin Gallate

Epigallocatechin-3-gallate (EGCG) is a flavan-3-ol or a polyphenolic catechin mainly found in green tea (*Camellia sinensis*); its molecule bears numerous hydroxyl groups, including one esterified as gallate in position 3 of its main catechin scaffold which provides its strong antioxidant and chelating properties [186]. It exhibits a wide range of pharmacological activities, including anti-inflammatory, cardioprotective, and antitumor [187]; in terms of anticancer effects, EGCG alters tumor angiogenesis, apoptosis and metastasis in numerous cancer cells by modulating multiple signaling pathways such as NF-κB, MAPK, PI3K/Akt, and nuclear factor erythroid 2-related factor 2 (Nrf2), thus being able to act as a chemopreventive and chemotherapeutic drug.

**Melanoma-specific mechanisms**

EGCG exerts antiproliferative and anti-metastatic effects in melanoma cells mainly through the inhibition of several key inflammatory and survival signaling pathways. Specifically, EGCG is able to inhibit the activity of tumor necrosis factor receptor-associated factor 6 E3 ubiquitin ligase thus resulting in the suppression of the TAK1/IκBα/NF-κB signaling pathway which is essential in tumor cell survival, inflammation and metastatic development [188]. EGCG also interferes with the activation of inflammasomes, mainly the NLRP1 complex, causing the in vitro and in vivo inhibition of caspase-1 activation and IL-1β secretion [189]. Collectively, it inhibits the pro-inflammatory signaling, thus contributing to the suppression of microenvironmental mechanisms that promote tumor progression.

**Figure 8:**
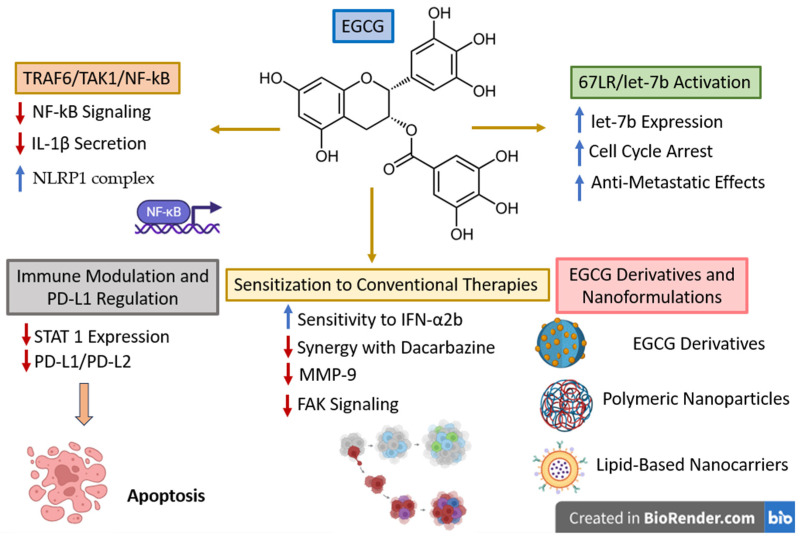
The sensitizing role of epigallocatechin gallate in melanoma immunotherapy. ↓—decrease/downregulation; ↑—increase/upregulation; Other arrows—leads to/generates (Created in BioRender. Oana Bătrîna. (2026) https://BioRender.com/8w99koc).

In addition to its direct inhibitory effects, EGCG also exerts epigenetic and post-transcriptional effects, such as the upregulation of the tumor suppressor microRNA-let7b through the activation of the 67 kDa laminin receptor (67LR) pathway [190]. Consequently, EGCG alters the downstream mechanisms responsible for cell cycle control, cell proliferation and metastasis thus inhibiting melanoma progression (Figure 8).

**Immune-related signaling and PD-L1 regulation**

EGCG has also been reported to alter several immune-related pathways such as the JAK/STAT signaling cascade that results in the subsequent downregulation of IFN-γ-induced PD-L1 and PD-L2 immune checkpoint ligands in melanoma cells [191]. These effects occur through the inhibition of STAT1 gene expression and STAT1 phosphorylation that trigger the downregulation of the PD-L1/PD-L2 transcriptional regulator IRF1 in both human and mouse melanoma cells. In vivo, the same study reported that EGCG acted through CD8+ T cells with similar inhibitory effects as the anti-PD-1 therapy. However, in terms of underlying molecular mechanisms, unlike the anti-PD-1 agents that block the PD-1/PD-L1 interaction, EGCG inhibits the JAK/STAT signaling and PD-L1 expression in tumor cells, thus leading to T cells re-activation. The authors concluded that EGCG may be considered an alternative anti-melanoma treatment able to target the PD-L1/PD-L2-PD-1 axis. However, these findings are still limited to preclinical assays with evidence indicating the indirect modulation of immune-related pathways instead of direct evidence regarding the immune checkpoint blockade or clear synergy with clinically approved immunotherapies.

**Sensitization to conventional therapies**

EGCG was investigated as a sensitizing agent in combination with other drugs; for example, EGCG increases the sensitivity of melanoma cells to interferon-α2b through apoptosis mediated by Fas (CD95) and inhibited the NF-κB signaling which is closely related to interferon resistance, finally inducing interferon-induced cell death [192]. In a similar manner, EGCG exhibited synergistic effects in combination with dacarbazine, leading to the inhibition of tumor invasion and metastasis through the inhibition of MMP-9 activity and focal adhesion kinase (FAK) signaling [193]. Nonetheless, these sensitizing effects have occurred in chemotherapy and cytokine-based therapies and were not yet identified in the immune checkpoint blockade.

**Immune checkpoint relevance**

Although, as mentioned above, EGCG was revealed to downregulate PD-L1 expression via the inhibition of JAK/STAT signaling, this effect alone does not represent a functional immune checkpoint blockade. Also, there is no current data regarding a direct synergy between EGCG and clinically approved immune checkpoint inhibitors (e.g., anti-PD-1, anti-PD-L1 or anti-CTLA-4 therapies) in melanoma. Moreover, EGCG was not revealed to overcome essential resistance mechanisms to the checkpoint blockade, such as defects in interferon signaling or antigen presentation. Therefore, one may state that EGCG indeed alters certain signaling pathways relevant in immune evasion, but its significance as immunotherapy sensitizer in melanoma is yet to be established.

**Chemical derivatization strategies and nanoformulation approaches**

Several chemical derivatization strategies have been proposed to overcome EGCG’s intrinsic pharmacokinetic limitations such as low chemical stability under physiological conditions, fast phase II metabolism, and poor membrane penetration that significantly reduce its systemic bioavailability. A comprehensive review was published by Dai et al. [194] that highlights the main approaches in this regard. Briefly, the authors mention esterification and acylation as widely explored strategies to increase a drug’s lipophilicity and membrane permeability; by substituting the hydroxyl groups with hydrophobic acyl chains, the resulting compound is able to interact more closely with membrane lipid bilayers and is less susceptible to oxidation. Of note, the substitution degree as well as its position must be carefully controlled since an extended chemical modulation may alter the pharmacophore drug constellation that is essential for its biological effects [194,195].

Recent studies further revealed that lipophilic derivatives such as the long-chain fatty acid conjugates benefit from an improved cellular uptake and retention due to their higher membrane affinity; in certain cases, the lipophilic derivatives may act as prodrugs that are able to release EGCG following intracellular hydrolysis [196].

Another direction is O-methylation which hinders the compound’s susceptibility to glucuronidation and sulfation by hindering the reactive catechol hydroxyl groups, thus optimizing its metabolic stability. Conversely, such modulations may also alter the drug’s redox activity and interactions with biological targets; therefore, a balance must be maintained between pharmacokinetic optimization and biological efficacy [194].

In the series of prodrug derivatization, peracetylated EGCG compounds have been synthesized where the hydroxyl groups were protected temporarily in order to increase its chemical stability, membrane permeability, and cellular uptake; inside the cell, the prodrug undergoes deacetylation and releases the active compound [197]. More recently, the research focused on the derivatization guided by structure–activity relationships studies; for example, the literature reported the selective modulation of the gallate moiety and regioselective hydroxyl protection in order to achieve adequate stability while maintaining its biological effects [198,199]. Although advances have been made, most derivatives were assessed in terms of optimized physicochemical properties and overall anticancer activity and not specifically in immuno-oncology. Currently, there is no evidence regarding the ability of EGCG derivatives to increase the modulation of the immune checkpoint or to overcome the resistant mechanisms that occur in melanoma immunotherapy.

Simultaneously, several nanoformulations were designed and developed in order to improve EGCG’s stability and derive efficacy. The same review by Dai et al. [194] describes the main nanosystems able to protect EGCG against chemical degradation, improve its solubility, and increase its systemic exposure. More recently, various types of nanoengineered delivery systems were designed and developed, including micelles, polymeric and lipid–polymer hybrid nanoparticles, metal-based nanoparticles, as well as functionalized nanocarriers able to improve the compound’s bioavailability and cellular uptake [200]. Of particular importance in melanoma treatment, transdermal delivery systems such as nanoethosomes have been revealed to be able to increase its skin penetration properties, thus facilitating its local accumulation and resulting in an improved therapeutic outcome [201]. However, it still remains to be established if such systems can indeed achieve tumor concentrations large enough to alter the immune checkpoint pathways in vivo; additionally, melanoma heterogeneity and EPR effect variability may limit the intratumor particle distribution [202].

Collectively, both the chemical modulation and the nanoformulation strategies are able to optimize the overall physicochemical and pharmacokinetic profile of EGCG, thus increasing tumor delivery; however, there are currently no specific data to clarify their influence on molecular mechanisms involved in the response to immunotherapy. Consequently, even if one manages to at least partially overcome its pharmacokinetic limitations, EGCG derivatives or optimized formulations have to be further assessed in terms of their ability to restore the interferon signaling, to improve antigen presentation or to overcome the resistance mechanisms identified in the immune checkpoint blockade in melanoma [54].

### 4.2. Polyphenols

#### Curcumin

Curcumin, also called diferuloymethane, is a natural polyphenol, extracted from *Curcuma longa* rhizome, that has been extensively studied and used as antioxidant [203], anti-inflammatory [204], antimicrobial [205], and anticancer agent [206] or as sensitizer to immunotherapy in cancer [207].

**Melanoma-specific mechanisms**

**Apoptosis signaling and oxidative stress**

Curcumin has primarily demonstrated direct antiproliferative and pro-apoptotic effects instead of confirmed immunotherapy sensitization. Bush et al. [208] conducted a study that showed curcumin-induced apoptosis; in melanoma cells, the results demonstrated that apoptosis was induced via the Fas receptor/caspase-8-dependent pathway and was not influenced by the p53 status. Additionally, recent evidence has revealed that curcumin induces oxidative stress, thus leading to melanoma cell death; curcumin increased ROS production, disrupted cell membrane, diminished glutathione, and activated the mitochondrial apoptosis pathways, thus inhibiting A375 melanoma cell proliferation [209]. In a similar way, Manica et al. [210] proved that curcumin stimulated apoptosis, lowered cell viability and migration, activated caspase-3, and increased ROS and nitrogen oxide levels in SK-MEL-28 melanoma cells. In SK-MEL-37 cells, curcumin also induced significant morphologic alterations and G0/G1 cell-cycle arrest, further supporting its direct cytotoxic activity against melanoma [211]. These findings indicate that curcumin indeed interferes with melanoma cell survival, but they mainly support direct antitumor activity rather than immune checkpoint sensitization [208,209,210,211].

**AKT/mTOR signaling, autophagy, and tumor progression**

Melanoma progression is inhibited by curcumin, which modulates the intracellular signaling pathways that are associated with tumor cell resistance and survival. Zhao et al. [212] demonstrated that in C8161 and A375 melanoma cells, curcumin triggered the inhibition of the AKT/mTOR pathway, the suppression of cell invasion and proliferation, the generation of G2/M cell-cycle arrest and the induction of autophagy both in vitro and in vivo. These results are mechanistically significant because the PI3K/AKT/mTOR pathway is intricately associated with metabolic adaptability, melanoma proliferation and therapeutic resistance. Furthermore, similarly to quercetin, the signaling pathway inhibition should be interpreted with caution since, although it may indirectly reduce the tumor associated immunosuppression, it does not clearly confirm that curcumin acts as a genuine sensitizer to immune checkpoint blockage. The evidence suggests that curcumin functions as a multitarget melanoma-active compound; nevertheless, it has not yet been validated as a sensitizer for melanoma immunotherapy [212].

**Immune checkpoint relevance: PD-L1/STAT1 signaling**

Xu et al. [163] conducted one of the most relevant studies on the topic and provided the most pertinent melanoma-specific evidence connecting curcumin to certain immune evasion pathways. Curcumin and apigenin were assessed in RPMI-7951, A375 and A2058 melanoma cell lines, as well as in the B16F10 murine melanoma model. In the study, the PD-L1 expression was stimulated by IFN-γ, and treatment with the two compounds suppressed IFN-γ-induced PD-L1 upregulation while also inhibiting STAT1 phosphorylation; however, apigenin proved more effective than curcumin in this regard. Specifically, curcumin partially restored the T cell-mediated cytotoxicity against melanoma cells, including PD-1-expressing Jurkat T cells, and the in vivo treatment was correlated with diminished tumor growth, lower Ki-67 and PD-L1 expression, and elevated CD8+ and CD4+ immune cell populations. These findings indicate that curcumin may reduce the adaptive immune resistance in melanoma by disrupting the IFN-γ/STAT-1/PD-L1 pathway. However, the results showed that apigenin was more potent than curcumin, and, notably, curcumin in direct combination with approved anti-PD-1/anti-PD-L1 was not evaluated in therapy. Therefore, even though curcumin exhibited clearly immune-related activity in melanoma, the current evidence remains insufficient to prove direct checkpoint sensitization in a therapeutically confirmed setting [163].

**Immune checkpoint relevance**

**Figure 9:**
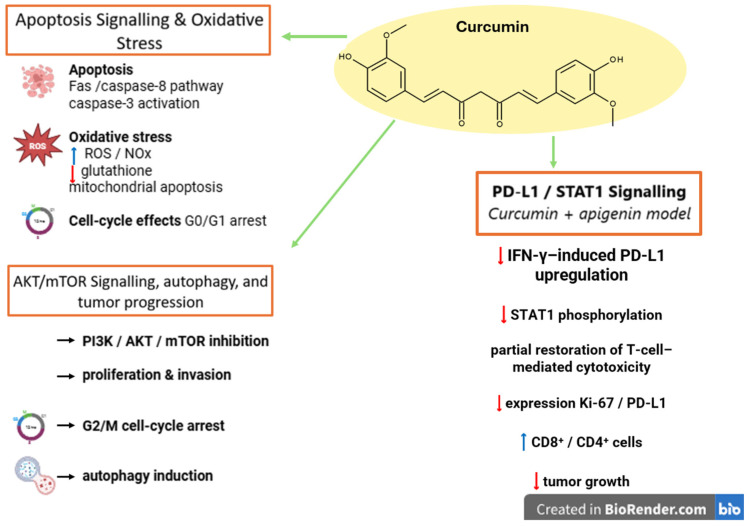
Schematic representation of the reported antiproliferative and immune-related effects of curcumin in melanoma, including apoptosis signaling, oxidative stress, AKT/mTOR inhibition, and PD-L1/STAT1 pathway modulation. ↓—decrease/downregulation; ↑—increase/upregulation; Other arrows—leads to/generates (Created in BioRender. Oana Bătrîna. (2026) https://BioRender.com/5yc0v31).

When considered collectively, the existing melanoma data suggests that curcumin can modulate the checkpoint-relevant biology through two interrelated mechanisms. The first one is based on diminishing the IFN-γ-induced PD-L1 expression by STAT1 signaling inhibition and the second consists of altering survival and inflammatory pathways that contribute to a tumor-promoting microenvironment [163,212]. These outcomes converge toward the conclusion that curcumin may enhance tumor vulnerability against immune-mediated cell death. Nonetheless, this concept remains incompletely validated because of the low number of melanoma research studies that directly test free curcumin in combination with clinically approved checkpoint inhibitors. Consequently, curcumin should be currently classified more as a compound with immune-modulatory activity relevant in melanoma rather than a validated immunotherapy sensitizer (Figure 9).

**Multimodal strategies: targeted nanodelivery and anti-PD-1 co-strategies**

Xiao et al. [213] reported an advanced approach linking curcumin with checkpoint-based immunotherapy by creating a dual pH-sensitive nanodrug that encapsulated curcumin and was decorated at the surface with anti-PD-1 antibodies. This platform was designed to maintain stability at a physiological pH and release curcumin more extensively in the mildly acidic tumor environment, thus integrating the checkpoint blockade with curcumin-mediated alterations of the tumor microenvironment. Further, it was noticed that the absorption of the nanodrug increased under acidic conditions in B16F10 melanoma cells and RAW264.7 macrophages. For the in vivo experiment, the formulation significantly inhibited tumor growth, extended overall survival, and diminished the production of NF-κB-associated immunosuppressive mediators, such as C-C motif chemokine ligand 22, IL-10 and TGF-β. However, the activity of this nanosystem simultaneously reflects the integrated performance of a multicomponent platform rather than that of curcumin alone. Therefore, the research endorses curcumin as a potentially useful component in an advanced immunotherapeutic formulation rather than as an independent checkpoint sensitizer.

**Resistance mechanism alignment**

At clinical level, notable resistance mechanisms to immune checkpoint blockade in melanoma encompass defective interferon signaling and compromised antigen presentation, including JAK1/JAK2 loss-of-function mutations and β2-microglobulin deficiency, which hinder the effective T-cell recognition and response. While curcumin modulates PD-L1-related adaptive resistance through the STAT1 pathway, there currently is no direct data indicating that it can restore or bypass these major resistance mechanisms in melanoma. This is a crucial limitation, since successful immunotherapy sensitization requires not only the regulation of checkpoint-related pathways but also clear activity against molecular aberrations that sustain primary and acquired resistance. Thus, the disparity between curcumin’s molecular potential and its clinical significance remains considerable [54,214].

**Pharmacokinetic limitations**

Curcumin exhibits pharmacokinetic flaws, like limited absorption, poor water solubility, rapid metabolism, and fast systemic elimination, in spite of its broad biological activity. In a frequently cited review, Anand et al. [215] presented phase I data in which it was specified that curcumin is well tolerated in humans at high oral doses, but due to its poor absorption, fast metabolism and systemic elimination, the plasma and tissue levels remain relatively low. Another review by Heger et al. [216] highlighted that curcumin undergoes considerable biotransformation, and the major barrier to its translation in oncology is its pharmacokinetic profile. Similarly to quercetin, the major issue is not solely its potency as an anticancer agent in vitro but whether physiologically significant concentrations can be obtained and maintained within tumors in vivo. This predicament is critical in melanoma immunotherapy, where the regulation of checkpoint mechanisms and immune cell interactions likely needs prolonged intratumor exposure instead of short-term or subtherapeutic systemic peaks.

**Delivery strategies and translational implications**

Due to these pharmacokinetic limitations, curcumin has been incorporated into a variety of advanced delivery systems designed to improve its solubility, enable its protection against metabolic degradation, prolong its circulation time, and increase tumor accumulation. In melanoma, Wang et al. [217] improved curcumin water dispersion by incorporating the phytocompound into biodegradable methoxy poly(ethylene glycol)-b-poly(L-lactide) (MPEG-PLA) nanoparticles. This nanoformulation curcumin/MPEG-PLA was associated with increased apoptosis, strong anti-angiogenic effects and stronger antitumor activity in A375 cells and B16 melanoma xenografts compared with the free curcumin [217]. The results strongly support the argument that the delivery system can actually enhance the pharmaceutical efficacy of curcumin in melanoma models. However, improved delivery cannot be automatically associated with efficient immunotherapy sensitization. Although the majority of available studies, like the pH-sensitive anti-PD-1 system [213] or the biodegradable MPEG-PLA nanoparticles [217], demonstrated an improved cell uptake, tumor inhibition, apoptosis or microenvironmental modulation, they have not proved conclusively that they could surpass clinically relevant resistance to checkpoint blockade. Collectively, the current delivery systems can be regarded solely as promising translational instruments; however, they are not yet definitive solutions to the immunotherapy-related constraints imposed by curcumin.

**Linking delivery to immunotherapy**

To date, the combination of curcumin-based nanoformulation as delivery systems and immunotherapy remains conceptually appealing, since nanocarriers might improve drug systemic exposure, boost tumor-specific accumulation and facilitate co-delivery of curcumin alongside antibodies or immunomodulators. Such an approach may theoretically allow curcumin to act at doses adequate to impact the PD-L1 expression, inflammatory cytokines, and immune cell recruitment. However, the existing data still fails to demonstrate that these systems can consistently convert non-responsive melanoma into checkpoint-sensitive disease. Furthermore, even if nanodelivery may reduce the pharmacokinetic disparity, it cannot independently overcome the mechanistic gap between preclinical and clinical immunotherapy sensitization. So, it is more appropriate to consider curcumin as a potential adjunct component of multimodal strategies rather than a self-contained sensitizer to checkpoint blockade [213,217].

**Chemical derivatization strategies**

Chemical derivatization is another way that can be used to overcome curcumin’s low bioavailability. Improved water solubility, chemical stability, membrane transport, or release behavior can be achieved by modifying its chemical structure. Safavy et al. [218] reported the PEG conjugation in order to generate water-soluble curcumin conjugates with improved cytotoxicity relative to the parent compound. Parvathy et al. [219] demonstrated that several curcumin–amino acid conjugates had strongly enhanced aqueous solubility compared to the natural curcumin. For curcumin–amino acid conjugates, Wan et al. [220] reported a significant antiproliferative activity and proteasome-inhibitory activity in cancer cell lines. Thus, it can be stated that semisynthetic modifications can mitigate curcumin’s limitations, but they still do not prove melanoma-specific pharmacokinetic superiority or immunotherapy relevance. Among ester-type prodrugs, the curcumin ester with diglutaric acid has been suggested as a highly water-soluble derivative with an adequate releasing profile; Muangnoi et al. [221] reported that this derivative revealed optimized oral bioavailability and, subsequently, increased biological effects compared to the parent drug. The limitations of such derivatizations occur for curcumin diethyl disuccinate in a study conducted by Bangphuni et al. [222], who found an inadequate oral bioavailability of the released curcumin due to its hydrolysis and first-pass metabolism in the gastrointestinal tract. Consequently, chemical derivatization represents a logical translational method, but it should not be considered as an automatically effective solution. Hence, certain derivatives clearly improve chemical stability or water solubility, but the critical question persists as to whether they can indeed ensure prolonged intratumor exposure and preserve curcumin’s immune-relevant efficacy at physiologically realistic levels. At this point, this issue remains unfortunately unsolved in the context of melanoma immunotherapy [218,219,220,221,222].

**Translational interpretation**

Collectively, the available literature suggests that curcumin has important anti-melanoma activity, consisting of apoptosis induction, oxidative stress-mediated cell death, inhibition of AKT/mTOR signaling, suppression of IFN-γ-induced PD-L1 expression, and regulation of inflammatory mediators. However, the evidence supporting curcumin as a genuine melanoma immunotherapy sensitizer remains moderate. The strongest melanoma-specific immune study demonstrated the PD-L1/STAT1 modulation and increased immune-mediated cell killing but did not approach a direct combination with approved checkpoint inhibitors. Additionally, the most advanced anti-PD-1-related study employed a multifunctional nanoplatform, making it impossible to discern the impact of free curcumin. Furthermore, neither the free curcumin nor its optimized formulations or derivatives have yet demonstrated efficacy in reversing significant melanoma resistance mechanisms such as JAK1/2 defects or β2-microglobulin loss. Therefore, curcumin should currently be considered a promising adjuvant candidate with melanoma-relevant immune activity and not yet a validated immunotherapy sensitizer. The future relevance of curcumin will depend on whether optimized delivery systems or semisynthetic derivatives can achieve pharmacologically significant exposure and prove consistent synergy with approved immune checkpoint inhibitors in melanoma-specific models [163,208,212,213,217].

### 4.3. Alkaloids

#### Berberine

Berberine is a naturally occurring quaternary ammonium isoquinoline alkaloid, characterized by its yellow color and bitter taste. It can be found in various salt forms, such as hydrochloride, sulfate, and phosphate, which have different degrees of water solubility [223]. *Berberis vulgaris*, *Berberis aristata*, *Berberis aquifolium*, *Hydrastus canadensis*, *Pellodendron chenins*, and *Coptis chinensis* are some of the varieties of plants from which it can be isolated. Berberine is well-known for its broad spectrum of biological and pharmacological activities and has been used traditionally in Ayurvedic and Chinese medicine. Berberine has attracted significant interest as a potential agent in multiple conditions, such as diabetes, depression, obesity, hypertension, inflammation and cancer [224].

**Melanoma-specific mechanisms**

**Growth inhibition, apoptosis, and cell-cycle effects**

Berberine exhibits a wide range of anticancer effects. Yet, in melanoma, the evidence still supports only direct antitumor effects, rather than activity as a sensitizing agent in immunotherapy. In a study, berberine inhibited cell viability and migration in human A375 melanoma cells, promoted apoptosis and altered cell-cycle regulatory miRNAs and proteins, such as CDK1, CDK2, cyclin D1 and cyclin A [225]. Palma et al. showed that berberine increased the expression of cytokines and proteins that are linked to apoptosis, further supporting its direct cytotoxic role in human melanoma cells [226]. These reported results indicate that berberine interferes with the survival of melanoma cells, but they do not, by themselves, establish immune checkpoint sensitization.

**Migration, invasion, and metastatic signaling**

The evidence specific to melanoma is particularly strong for the anti-metastatic effect of berberine; it reduced metastatic potential through AMPK activation, with downstream reduction in ERK activity and COX-2 protein expression in melanoma cells. These effects were supported both in vitro and in a pulmonary metastasis model in vivo [227]. In human A375.S2 melanoma cells and PLX4032-resistant A375.S2 cells, berberine also inhibited motility, migration, and invasion through direct effects on the FAK, urokinase plasminogen activator, and NF-κB signaling, together with changes in epithelial–mesenchymal transition- and metastasis-related proteins such as E-cadherin, N-cadherin, RhoA, p-FAK, p-AKT, p-ERK1/2, and urokinase plasminogen activator [228]. Collectively, these studies support a reproducible anti-invasive and anti-metastatic effect of berberine in melanoma models. However, these mechanisms remain more relevant to tumor progression and dissemination than to direct checkpoint sensitization.

**Immune checkpoint relevance**

It has been reported that berberine regulates the PD-1/PD-L1 axis, but the earliest direct evidence for this mechanism comes from non-melanoma models. For example, berberine decreased tumor-cell PD-L1 expression, facilitated antitumor immunity and promoted PD-L1 degradation in lung cancer and Lewis tumor models by inhibiting the deubiquitinating activity of COP9 signalosome 5. This further results in enhanced T-cell immunity and reduced immunosuppressive MDSC and Treg populations [229]. This data, on its own, remains supportive rather than demonstrating effects on melanoma, but it is highly relevant mechanistically.

**Melanoma immunotherapy sensitization**

**Figure 10:**
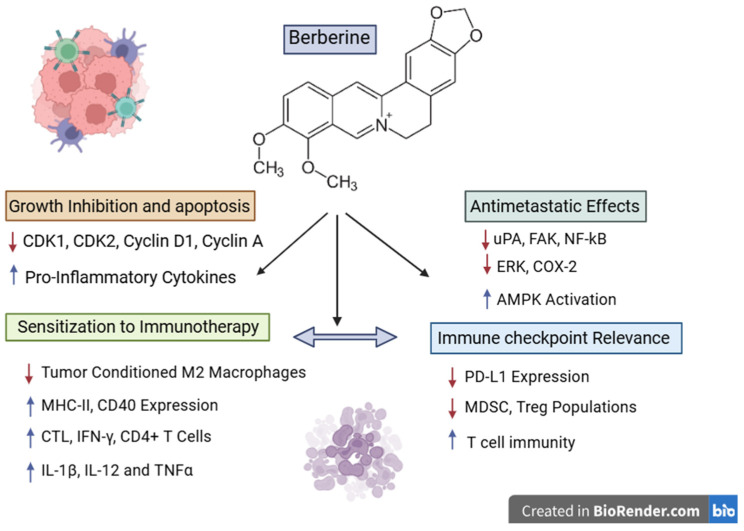
Mechanism of berberine as a potential chemosensitizing and immunomodulatory agent in melanoma. ↓—decrease/downregulation; ↑—increase/upregulation; Other arrows—leads to/generates (Created in BioRender. Oana Bătrîna. (2026) https://BioRender.com/kw8dft6).

An immune sensitizing mechanism specific to melanoma was described in a B16F10 murine melanoma model in which berberine skewed immunosuppressive macrophages associated with the tumor toward an inflammatory phenotype. Berberine inhibited the IL-6/STAT3/IL-10 axis and reduced IL-10 and TGF-β release. It also prevented tumor-conditioned shifting of macrophages toward an M2-like phenotype, increased MHC-II and CD40 expression, enhanced IL-1β, IL-12, and TNFα release, and promoted CTL activity and IFNγ-producing CD4+ T cells (Figure 10). In vivo, berberine reduced tumor volume and weight and also increased the frequency of M1-like macrophages in melanoma-bearing mice [230]. These results represent one of the strongest arguments for berberine as an immune-modulatory adjuvant in melanoma because it directly addresses the immunosuppressive tumor microenvironment rather than merely tumor cell cytotoxicity. Still, this study supports immune relevance more strongly than it proves checkpoint sensitization, as it did not test berberine in combination with approved checkpoint inhibitors.

A second and more directly relevant melanoma study showed that berberine can sensitize the immune checkpoint blockade through the NAD(P)H quinone oxidoreductase 1 (NQO1) inhibition and ROS activation. In syngeneic melanoma models, berberine induced immunogenic cell death with the release of ATP, HMGB1, and calreticulin, promoted dendritic-cell maturation, and increased CD8+ and memory T-cell responses. Importantly, berberine enhanced the efficacy of anti-PD-1 and anti-CTLA-4 therapy and improved survival compared to the checkpoint blockade alone [231]. Among the currently available berberine data, this is the clearest evidence that the compound can function as a melanoma immunotherapy sensitizer rather than only as a direct cytotoxic agent. Still, the evidence remains preclinical and limited to a small number of models.

**Resistance mechanism alignment**

Defective interferon signaling and loss of antigen presentation, particularly through JAK1/2 inactivation and β2-microglobulin loss, are the clinically established resistance mechanisms to immune checkpoint blockade in melanoma. A clinically relevant sensitizer should restore or bypass at least a part of these pathways [232]. Current studies on berberine support macrophage repolarization, immunogenic cell death, dendritic-cell activation and improved T-cell priming, but there is still no direct evidence that berberine reverses JAK1/2-dependent interferon resistance or β2-microglobulin loss in melanoma. Consequently, an important gap remains between promising preclinical immune effects and the key resistance mechanisms responsible for the failure of checkpoint blockade in patients.

**Pharmacokinetic limitations**

Berberine’s clinical use is still limited by several well-known pharmacokinetic challenges, such as poor oral bioavailability, extensive first-pass metabolism, and low systemic exposure. Specific reviews on the issue show that poor intestinal absorption and high first-pass elimination significantly restrict free berberine exposure in vivo [233,234]. These liabilities are important when considering melanoma immunotherapy, because it may be difficult to achieve biologically relevant intratumor concentrations by using free berberine alone. Accordingly, as with other phytochemicals, the translational dilemma is not only whether berberine works in vitro but whether sufficient tumor exposure can be sustained in vivo in order to modulate immune-relevant pathways.

**Delivery strategies and translational implications**

Because of these pharmacokinetic barriers, the most convincing data on berberine use in anti-melanoma immunotherapy come from experiments that either reprogram the tumor microenvironment or couple berberine to a defined immune-modulating strategy. The macrophage-repolarization study shows that berberine can restore inflammatory signaling in the tumor microenvironment through STAT3 inhibition [230], whereas the NQO1/ROS study indicates that berberine can enhance the checkpoint blockade by inducing immunogenic cell death [231]. These findings provide stronger evidence than a simple revision of antiproliferative effects because they respond directly to the need for mechanistic depth and therapeutic relevance. At the same time, both studies remain at the preclinical level and neither has yet proven that berberine is able to overcome the full spectrum of clinically relevant resistance seen in human melanoma.

**Chemical derivatization strategies**

Another strategy to overcome the pharmacokinetic limitations of berberine is its chemical derivatization, which aims to improve its membrane permeability, metabolic stability and systemic exposure by modifying the parent isoquinoline scaffold. This approach is particularly relevant because berberine exhibits poor oral bioavailability and substantial first-pass limitation, which significantly restricts in vivo exposure of the free compound [233,234].

One of the best-known examples is 8,8-dimethyldihydroberberine, a reduced B derivative reported to show improved bioavailability and oral efficacy in preclinical models when compared with berberine [235]. This supports the concept that structural modification of the berberine scaffold can improve the in vivo performance of the compound rather than relying exclusively on external delivery systems. In addition, a berberine prodrug strategy has also been reported, with the design of berberrubine (an active metabolite of berberine) specifically intended to improve the pharmacokinetic parameters of berberine [236].

From a translational perspective, these derivatizations bear high significance since they directly address one of berberine’s major barriers to clinical application. However, the currently available derivatives have been studied mainly in the context of pharmacokinetic optimization and non-melanoma disease models, not as validated melanoma immunotherapy sensitizers. Therefore, chemical derivatization should be regarded as a rational and promising strategy to enhance the drug in vivo exposure but not yet as evidence that berberine derivatives can overcome clinically relevant resistance to checkpoint blockade in melanoma.

**Translational interpretation**

Collectively, the available literature indicates that berberine has important anti-melanoma activity, including inhibition of cell proliferation, induction of apoptosis, suppression of cell migration and invasion, AMPK-dependent anti-metastatic activity, macrophage repolarization, and enhancement of immune-mediated tumor control [225,226,227,228,230,231]. The strongest evidence for melanoma-relevant immunotherapy now comes from the macrophage-centered remodeling of the tumor microenvironment and from the NQO1-dependent induction of immunogenic cell death, rather than from the PD-L1 modulation alone. Therefore, berberine can reasonably be classified as a promising adjuvant candidate in combination therapies given its melanoma-relevant immune activity and, compared with several other phytochemicals, has somewhat stronger preclinical support as an immunotherapy sensitizer. However, it still cannot be considered a validated melanoma checkpoint sensitizer until its effects are confirmed in broader melanoma models and aligned with clinically relevant resistance mechanisms.

## 5. Plant-Derived Compounds: Indirect or Emerging Sensitizers in Melanoma

### 5.1. Indirect or Emerging Sensitizers in Melanoma

The compounds discussed below demonstrate melanoma-relevant anticancer or immune-modulatory activities; however, existing evidence for the direct sensitization to immune checkpoint blockade in melanoma is scarce, indirect, or dependent on specific tailored delivery systems or methods. To prevent overinterpretation, these compounds are more accurately categorized as indirect or emerging sensitizers instead of validated melanoma immunotherapy sensitizers. In the majority of cases, the literature supports one or more of the following: direct cytotoxicity against melanoma, inhibition of invasion or stemness, regulation of macrophage polarization or inflammatory signaling, or corroborative evidence from non-melanoma immunotherapy models. However, direct combinations involving approved checkpoint inhibitors in melanoma are limited, and the alignment with clinically recognized resistance mechanisms (defective interferon signaling, loss of antigen presentation) remains inadequately substantiated [54,237].

#### 5.1.1. Kaempferol

Kaempferol, a naturally occurring flavonol that displays a polyphenolic structure, has proven antioxidant, anti-inflammatory and anticancer properties. It has been demonstrated that, in cancer cells, kaempferol induces apoptosis and cell cycle arrest and suppresses proliferation and metastasis by modulating pathways like NF-κB, MAPK, and PI3K/Akt/mTOR [238,239]. The existing evidence in melanoma remains insufficient but indicates direct cytotoxicity. Yang et al. [240] demonstrated that in A375 human melanoma cells, kaempferol generated apoptosis and cell-cycle arrest, as well as inhibited migration via the PI3K/Akt/mTOR pathway. Even if this study sustains directly anti-melanoma activity, it does not produce direct proof for checkpoint-related sensitization. Additionally, kaempferol hindered melanoma metastasis by inhibiting aerobic glycolysis through the disruption of hexokinase 2/voltage-dependent anion channel 1 interaction [241]. The presented mechanism is biologically significant because of the potential impact of metabolic reprogramming on tumor progression. Nevertheless, the existing data does not prove a direct improvement of the checkpoint blockade.

Collectively, kaempferol must be regarded as a flavonol with activity against melanoma and with indirect immune relevance. Its significance currently resides in the direct antitumor and anti-metastatic effects and not in a validated immunotherapy sensitization.

#### 5.1.2. Resveratrol

Resveratrol is a natural stilbene polyphenol found in grapes, berries, peanuts, cocoa, and *Reynoutria japonica*. In melanoma, the strongest evidence supports direct antiproliferative and pro-apoptotic effects. In MV3 and A375 human melanoma cells, resveratrol triggered apoptosis through the inhibition of the ERK/PKM2/Bcl-2 pathway, with increased p53 expression and caspase activation [242]. An additional study, conducted by Heo et al. [243] on A375SM melanoma cells, identified ROS generation, endoplasmic reticulum stress, p38 and p53 activation, and apoptosis together with cell-cycle arrest. In murine B16 melanoma cells, resveratrol decreased proliferation through the inhibition of the SHCBP1–ERK1/2 axis, which led to G1/S arrest and upregulated p21 expression [244]. Niles et al. [245] also demonstrated growth inhibition and apoptosis in A375 and SK-MEL-28 melanoma cells.

In terms of melanoma stemness and therapy resistance, resveratrol is considered significant. In A375 malignant melanoma stem-like spheroids, resveratrol boosted the response to all-trans retinoic acid by decreasing the expression of CD271, CD133, ABCG2 and octamer-binding transcription factor 4 (OCT4), while increasing SRY-box transcription factor 9 (SOX9) and reducing SOX10 consistent with a shift toward a more differentiated phenotype. The outcome involved an association with decreased DNA methyltransferase 1, re-expression of retinoic acid receptor beta (RARβ), suppression of enhancer of zeste homolog 2 (EZH2) and B cell-specific Moloney murine leukemia virus integration site 1 (BMI-1), and higher levels of p16, p19 and p21. In addition, pretreatment increased subsequent sensitivity to docetaxel [246]. The findings are significant since they reveal a clear connection between resveratrol and a biologically relevant region that is associated with therapeutic resistance. However, it is not proven to be a direct checkpoint sensitization mechanism.

The proof that connects resveratrol to immune checkpoint biology is more limited and is primarily derived from non-melanoma types of cancer. Resveratrol promoted the intracellular accumulation of PD-L1 and enhanced antitumor T-cell immunity in breast cancer by interfering with PD-L1 glycosylation and dimerization [247]. In contrast, the research of Lucas et al. [248] on colorectal and breast cancer proved that resveratrol, free or combined with piceatannol, could enhance PD-L1 expression by activating the NF-κB signaling pathway via histone deacetylase (HDAC)3/p300. The results of these two studies indicate that resveratrol regulated PD-L1 in a context-dependent manner, rather than in a uniformly suppressive direction.

Jia et al. [249] developed a dual-responsive polyplex system that delivers resveratrol together with PD-L1-targeting siRNA, thus generating a more direct link between resveratrol and immunotherapy. This platform improved antigen presentation-related signaling, dendritic-cell and CD8+ T-cell responses, reduced glycolysis, increased oxidative phosphorylation, and enhanced PD-L1 silencing in B16F10 melanoma and CT26 carcinoma cells. In comparison to each component alone, the combined therapy resulted in enhanced overall survival, reduced lung metastasis, and stronger tumor suppression in vivo. This study can be considered one of the strongest examples of a delivery-based immunotherapy strategy that involves resveratrol. However, the downfall is that the study investigated the effect of a multicomponent platform, the effect being the result of all the involved components, not only the unbound resveratrol alone. Dourado et al. [250] also investigated the specific delivery in A375 melanoma cells of an *Attalea funifera* oil containing resveratrol-loaded organogel nanoparticles. The nanoparticle formulation exhibited an optimized pharmaceutical performance but did not specifically validate immunotherapy sensitization.

From a translational perspective, resveratrol remains restricted by rapid metabolism, low free systemic exposure, and low circulation time [251]. An increased exposure could be achieved through chemical derivatization, like O-methylation, in order to generate pterostilbene and to create amino acid carbamate prodrugs [252,253], although these molecular modifications did not result in a validated sensitizer for melanoma immunotherapy. Generally, resveratrol is mostly regarded as a promising indirect or emerging sensitizer in melanoma, with evidence indicating a direct anticancer effect, modulation of stemness, and engineered immunotherapy combinations.

#### 5.1.3. Ursolic Acid

Ursolic acid is a pentacyclic triterpene found in apples, berries, lavender, thyme, rosemary, and other medicinal/edible plants. In melanoma, the available evidence supports direct cytotoxic effects, especially through the induction of apoptosis and cell-cycle arrest. Manu et al. [254] demonstrated that in B16F10 melanoma cells, ursolic acid induced apoptosis via p53 and caspase-3 activation and by the suppression of NF-κB-mediated activation of anti-apoptotic Bcl-2. Moreover, according to Harmand et al. [255], in human melanoma M4Beu cells, ursolic acid induced apoptosis through the mitochondrial intrinsic pathway, with mitochondrial membrane potential disruption and caspase-3 activation.

Certain reports indicate immunological significance in melanoma, involving the reduction in inflammatory cytokine levels and enhanced NK-cell activity in tumor-carrying mice; however, the evidence remains sparse and merely preclinical [254].

Supportive non-melanoma research implies that ursolic acid formulations might have broader immune-modulatory potential. Among various cancer models, Zang et al. [256] demonstrated that ursolic acid-loaded liposomes can be associated with the reduction in Treg cells and myeloid-derived suppressor cells. Furthermore, ursolic acid-enriched Kudingcha extract has been reported by Xu et al. [257] as a stimulating agent in bacteria-mediated cancer immunotherapy, partly due to its anti-angiogenic effects that involve VEGF receptor 2 downregulation. Still, these results are indirect evidence and cannot be extrapolated to melanoma checkpoint blockade. Therefore, at this point, ursolic acid can be classified as an indirect candidate with suggestive immunomodulatory significance, rather than a validated melanoma immunotherapy sensitizer.

#### 5.1.4. Betulinic Acid and Betulin

Betulinic acid is a lupane-type pentacyclic triterpene derived from the bark of white birch, which remains one of the most widely investigated triterpenes in melanoma. Early medicinal chemistry work showed that betulinic acid and related derivatives inhibited growth in cultured human melanoma cells [258]. A later primary melanoma study demonstrated that betulinic acid strongly and consistently suppressed growth and colony-forming ability across multiple human melanoma cell lines and showed additive interactions with ionizing radiation [259]. More recent studies on betulin derivatives also support anti-melanoma activity in metastatic melanoma models [260]. Taken together, the primary melanoma literature supports direct anti-melanoma cytotoxicity and combination potential with other treatments, while the evidence for immune sensitization remains indirect. Non-melanoma studies suggest effects on macrophage polarization, inflammatory signaling, and combination regimens, but these should be treated only as supportive data rather than as direct evidence for melanoma immunotherapy. Thus, betulinic acid is best viewed as a melanoma-active triterpene with indirect immune relevance, not as a validated checkpoint sensitizer.

Betulin is the natural precursor of betulinic acid and is generally less potent as an anticancer agent; it still remains relevant as a scaffold in medicinal chemistry. The currently existing melanoma evidence is stronger for betulin derivatives than for betulin itself, with recent studies supporting their anti-melanoma activity in metastatic melanoma models [260]. At the same time, there is no direct evidence of betulin as an immune sensitizer in melanoma. Accordingly, betulin stands mainly as a promising scaffold for chemical derivatizations rather than as a standalone immunotherapy sensitizer.

#### 5.1.5. Matrine

Matrine is a tetracyclic quinolizidine alkaloid that can be isolated from *Sophora flavescens* and related species. It has a wide spectrum of pharmacological effects, including anti-inflammatory, antiviral, immunomodulatory, and anticancer effects [261]. It has been reported that matrine and its derivatives modulate several oncogenic pathways in cancer models, such as Wnt/β-catenin, MAPK/ERK, and PI3K/AKT/mTOR. They also suppress proliferation, promote apoptosis and inhibit the epithelial–mesenchymal transition [262].

In melanoma, matrine has been shown to inhibit proliferation and invasion and to induce apoptosis in A375 and SK-MEL-2 cells. These effects were linked, at least in part, to the downregulation of miR-19b-3p and the upregulation of phosphatase and tensin homolog (PTEN), a tumor suppressor highly relevant in both melanoma progression and tumor–immune interactions [263]. Because PTEN loss contributes to immune evasion and reduced response to immunotherapy [264], the ability of matrine to restore PTEN expression is mechanistically interesting. Beyond this, however, the argument for immune sensitization rests mainly on the plausibility of the pathway rather than on direct checkpoint-related evidence. Therefore, to date, matrine is best described as a mechanistically interesting but weakly validated indirect sensitizer whose relevance derives mainly from melanoma activity and PTEN-related biological plausibility rather than direct immunotherapy evidence.

#### 5.1.6. Sulforaphane

Sulforaphane is an aliphatic isothiocyanate generated from glucoraphanin, a glucosinolate abundant in cruciferous vegetables such as broccoli, cauliflower, cabbage, and Brussels sprouts, by myrosinase-dependent hydrolysis, and it is rapidly absorbed and metabolized, mainly through glutathione conjugation and the mercapturic acid pathway. Although it is often described as highly bioavailable, systemic exposure varies substantially depending on the food matrix and the presence of active myrosinase, which is important when considering translational relevance [265,266].

Sulforaphane has broad anticancer activity through the modulation of several interconnected pathways, including the inhibition of NF-κB, STAT3, PI3K/AKT, and MAPK signaling, the activation of the Nrf2–antioxidant response element axis, and the regulation of epigenetic processes such as HDAC and DNMT activity. Through these mechanisms, it can promote apoptosis and suppress invasive and stemness-associated cell phenotypes [267]. However, in melanoma, the strongest evidence still supports direct antitumor activity rather than validated immunotherapy sensitization.

Sulforaphane caused apoptosis in highly metastatic B16F10 melanoma cells by activating caspase-3 and caspase-9, increasing Bax and p53, and lowering the levels of Bcl-2, Bid, NF-κB, and caspase-8 [268]. In another B16F10 metastasis model, sulforaphane decreased the burden of pulmonary metastasis and blocked pathways related to invasion, which supports its role as an anti-invasive and anti-metastatic agent in the progression of melanoma [269]. Sulforaphane also enhanced cell-mediated immune responses in B16F10 melanoma-bearing mice, including increased NK-cell activity, increased antibody-dependent cellular cytotoxicity, and improved lymphoproliferative responses, together with reduced tumor-associated inflammatory cytokines [270]. These studies indicate that sulforaphane exerts important anti-melanomal effects, but they do not directly demonstrate sensitization to immune checkpoint blockade.

The combination of sulforaphane with other compounds further supports its significance in melanoma. The combination of sulforaphane and quercetin suppressed melanoma the progression more effectively than either agent alone, partly through downregulation of MMP-9 expression, thus suggesting that sulforaphane can participate in multi-agent anti-melanoma strategies [271]. Evidence linking sulforaphane more directly to immunotherapy comes mainly from non-melanoma cancers and should therefore be regarded as supportive rather than melanoma-validating. In dendritic-cell models exposed to pancreatic tumor-derived antigens, sulforaphane enhanced T-cell stimulatory capacity by modulating co-stimulatory and inhibitory molecules, including CD80, CD83, and B7-H1/PD-L1. Mechanistically, it inhibited JAK/STAT3 signaling, downregulated B7-H1 expression, and altered microRNA profiles, thereby promoting T-cell activation [272]. These data increase the plausibility that sulforaphane could support antitumor immunity, but they remain indirect with respect to the melanoma checkpoint blockade.

Collectively, sulforaphane should be classified as an indirect or emerging sensitizer in melanoma and not as a validated melanoma immunotherapy sensitizer. The melanoma-specific literature supports apoptosis induction, reduced metastatic behavior, and partial immune modulation [268,269,270], while supportive non-melanoma studies suggest that sulforaphane can influence PD-1/PD-L1-related signaling and dendritic-cell function [272]. However, no direct studies have yet shown that sulforaphane sensitizes melanoma to approved immune checkpoint inhibitors, and the current evidence does not demonstrate reversal of clinically relevant resistance mechanisms such as JAK1/2-dependent interferon defects or β2-microglobulin loss. Accordingly, sulforaphane is best presented as a biologically plausible adjunct candidate whose translational relevance will depend on future melanoma-specific combination studies with checkpoint blockade.

**Concluding perspective on indirect or emerging sensitizers**

**Figure 11:**
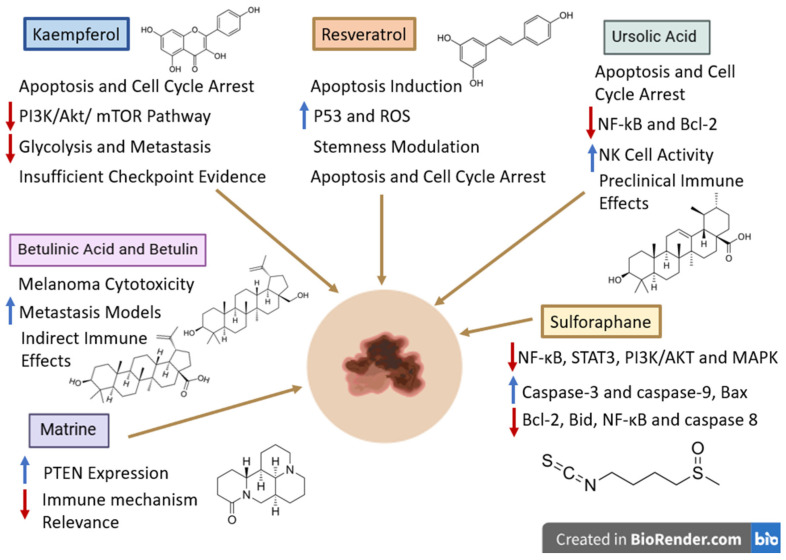
Indirect and emerging sensitizers in melanoma: mechanistic insights and immunotherapy relevance. ↓—decrease/downregulation; ↑—increase/upregulation; Other arrows—leads to/generates (Created in BioRender. Oana Bătrîna. (2026) https://BioRender.com/3a50i5v).

Overall, the compounds examined in this section should not be interpreted as confirmed sensitizers for melanoma immunotherapy. They constitute a heterogeneous assembly of indirect or emerging candidates whose relevance originates from melanoma-specific cytotoxicity, reduction in stem-like or therapy-resistant characteristics, modulation of macrophage polarization or inflammatory signaling, alteration of antigen presentation-related pathways, or corroborative evidence from non-melanoma immunotherapy research. Their translational relevance is limited by the shortage of direct checkpoint-combination studies in melanoma, insufficient correlation with clinically established resistance mechanisms such as JAK1/JAK2 or β2-microglobulin defects, and, in several cases, undesirable pharmacokinetic properties [54,237]. Consequently, these compounds are optimally suited as preclinical adjuvant candidates requiring additional investigation, rather than as validated sensitizers in clinical applications (Figure 11).

## 6. Discussion

All the evidence presented in this review suggests that plant-derived compounds have a heterogeneous position as potential sensitizers in the melanoma immunotherapy landscape. Although all the discussed phytocompounds have been associated with antitumor or immunomodulatory effects, their actual relevance and the degree to which these effects are relevant to immune checkpoint inhibition vary substantially. By comparatively reading and interpreting the available literature data, no uniform binary classification (active vs. inactive sensitizers) of these compounds can be formulated but rather provide a gradient and nuanced view of their role as melanoma immunotherapy sensitizers. The data show different levels of biological, mechanistic and translational relevance, with only a limited number of compounds showing a direct improvement of response to checkpoint inhibitors in melanoma.

To better understand and interpret it, the evidence can be organized into three comparative tiers: (1) compounds with demonstrated antiproliferative/pro-apoptotic and immune-relevant effects, such as checkpoint-associated signaling modulation, activation of innate immune pathways, enhancement of cytotoxic immune responses, or remodeling of the TME in melanoma-based studies; (2) compounds with demonstrated specific anti-melanoma effects, such as suppression of proliferation, invasion, migration, oxidative stress, or oncogenic signaling, but with indirect immune-relevant findings and without a clear demonstration of sensitization to immune checkpoint therapy; (3) compounds with a proposed sensitizing role based on evidence from other tumor types, in which the data is mechanistically informative but not demonstrated in melanoma-specific settings.

This review shows that the quality of the evidence is not solely determined by the reported mechanism but by the experimental depth at which that mechanism is validated. Some compounds are supported by multiple independent studies in melanoma models, including in vivo findings and immune-associated endpoints, while others are supported mainly by isolated in vitro observations, docking data, or data from unrelated cancers. Thus, the difference between the mechanistic mention and mechanistic support is the basis of an objective synthesis. Therefore, the strongest candidates are not necessarily those with the longest list of reported targets but those for which the same immune-relevant themes are repeated across melanoma-specific systems and levels of analysis. Thus, reproducibility of mechanistic direction is more informative than mechanistic abundance alone.

After the overall assessment of the studies presented, several convergent mechanistic points were observed. Firstly, many phytocompounds act on the same intracellular pathways, such as STAT 3, NF-κB, PI3K/Akt, MAPK, and redox-sensitive signaling pathways. These pathways collectively modulate inflammatory cytokine signaling, cellular redox balance, immune-cell recruitment, immune evasion, and ultimately melanoma cell survival. Hence, despite their chemical diversity, their immunobiological effects seem to be clustering around common signaling architecture, rather than around entirely different compound-specific mechanisms. Additionally, it seems that even though many of these phytocompounds do not seem to act as direct checkpoint-targeted agents, they are, however, able to influence the TME and the background in which the immune checkpoint inhibitors act. Specifically, they can influence macrophage polarization, dendritic-cell activation, inflammatory cytokines, oxidative stress and T cell-associated responses; all these effects might increase tumor immunogenicity, attenuate immunosuppressive signaling, and reshape the local immune milieu so that response to checkpoint blockade improves. This aspect proposes that the phytocompounds’ value may reside in their ability to modulate the tumor immune environment rather than in reproducing the pharmacology of checkpoint blockade. Lastly, only a small number of studies were able to demonstrate a direct effect of certain phytocompounds on checkpoint-related endpoints, such as synergy with immune checkpoint inhibitors, PD-L1 modulation, or increase in cytotoxic immune activity in relevant models.

In this comparative context, flavonoids emerge as the most abundant and mechanistically versatile class of compounds. Quercetin, luteolin, EGCG and kaempferol appear frequently in melanoma studies that report modulation of cell survival, inflammatory transcription factors, oxidative stress, cytokine signaling, and, in some cases, checkpoint-associated or innate immune pathways. Quercetin is supported by data that is melanoma-specific and immune-oriented, although the currently available evidence still does not validate it as an established immunotherapy sensitizer, whereas the other flavonoids are only characterized by general anticancer activity or by indirect data supporting their sensitizing role. The polyphenols are another prominent group that has a different comparative profile; the studies revealed anti-inflammatory, anti-invasive, antiproliferative effects and TME effects with plausible links to immune-associated pathway regulation. However, the studies do not provide a direct link between the general anticancer activity and checkpoint sensitization; the gap between these two is more pronounced in this group compared to flavonoids. This class is relevant from a mechanistic standpoint but less consolidated judging from a melanoma-oriented immunotherapy endpoint view. Similar observations also emerge for isothiocyanates, triterpenes and alkaloids. These groups have high biological activity in melanoma settings and significantly expand the mechanistic spectrum; however, with the exception of berberine, their melanoma-specific checkpoint-oriented validation is still limited and the presently available literature only suggests their potential of becoming relevant sensitizers.

To support a more structured comparison, we synthesized the reviewed compounds comparatively, according to melanoma-specific evidence, immune relevance in melanoma, checkpoint-oriented significance and mechanistic reach. This semi-quantitative framework is designed to distinguish candidates of higher priority from exploratory compounds and to provide an objective basis for cross-compound interpretation (Table 1).

The broad synthesis of the studies revealed a strong emphasis on the phytocompounds’ multitarget activity, which influences survival pathways, tumor proliferation, apoptosis modulation, inflammatory mediators, cell and TME interactions, oxidative stress, and checkpoint-related molecules simultaneously. This multiplicity is relevant in melanoma settings where immune escape does not depend on a single pathway. The ability to influence at the same time oncogenic signaling, inflammation, oxidative stress, stromal interactions and immune suppression give that compound, in theory, an increased advantage over highly restricted agents. However, at the same time, the literature suggests that this multitarget activity can complicate interpretation. It becomes challenging to determine which is the primary effect, which are downstream effects or which ones are context-dependent; hence, it is difficult to establish whether the checkpoint-associated changes are primary drivers of sensitization or just secondary consequences of a broader tumor-cell stress and signaling reprogramming. This multitarget behavior described for many phytocompounds should be viewed as a characteristic that increases biological interest, not as direct proof that each target can contribute equally to immunotherapy sensitization. All the presented studies suggest that the tumor-intrinsic and the immune-related effects should not be viewed as completely separate categories. Numerous studies show that some phytocompounds can alter immune-relevant pathways and, at the same time, can induce apoptosis, cell-cycle arrest, and suppression of oncogenic transcription factors or reduced cell migration. For example, as demonstrated for quercetin in melanoma models, tumor-cell stress may promote immunogenic signaling by increasing antigen release and presentation and by inducing DAMPs that favor dendritic-cell activation and T-cell priming; in parallel, inhibition of melanoma survival pathways may indirectly attenuate immunosuppressive signaling and contribute to TME reprogramming [98,109]. Consecutively, a clear distinction between a “direct anticancer activity” and “immune sensitization” can oversimplify these phytocompounds’ functions. Therefore, a more integrated view entails that they exert pleiotropic effects that simultaneously weaken the melanoma cells and reshape the local immune context, taking into account that the relative contribution of these distinct pathways differs from one compound to another.

**Figure 12:**
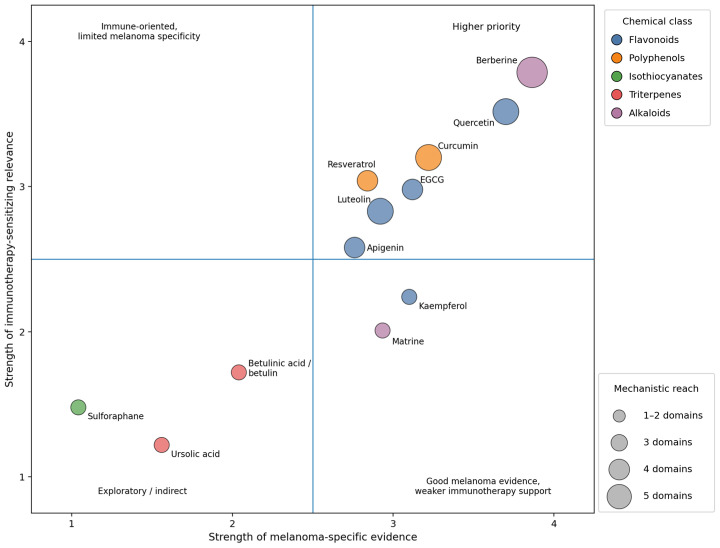
Comparative evidence map of plant-derived compounds discussed as potential sensitizers to immunotherapy in melanoma. Compounds were plotted based on the strength of melanoma-specific evidence (x-axis) and immunotherapy-sensitizing relevance (y-axis). The size of the bubble shows mechanistic reach, respectively, the number of major biological domains that have been reported for each compound. These include tumor-intrinsic signaling, checkpoint-associated signaling, innate immune activation, T cell-related effects, and TME modulation. Compounds in the upper-right quadrant are high-priority candidates with strong melanoma support and relevancy for immunotherapy. Compounds in the lower-left quadrant are still weakly substantiated for their melanoma-specific sensitizing role and are mostly supported by indirect or limited evidence. Melanoma-specific evidence was scored from 1 to 4 as follows: 1 (low): low evidence/absent or largely deduced from non-melanoma studies; 2 (limited): some melanoma evidence, present but mainly isolated in vitro tumor-cell data; 3 (moderate): multiple melanoma studies and/or at least one in vivo melanoma model; 4 (high): consistent melanoma-specific evidence including immune-relevant endpoints, in vivo support, or combination-oriented relevance. The immunotherapy-sensitizing relevance was scored from 1 to 4 as follows: 1 (low): mainly general anticancer and/or anti-inflammatory effects without a clear immunotherapy link; 2 (limited): with indirect immune relevance or checkpoint-associated mechanisms; 3 (moderate): some immune-relevant effects in melanoma or plausible checkpoint-oriented findings; 4 (high): direct evidence of checkpoint-related modulation, immune activation relevant to checkpoint response, or reported synergy with immune checkpoint blockade.

For a better understanding of the comparative hierarchy shown in Table 1, the same evidence framework was transformed into a more visually comprehensive two-dimensional evidence map (Figure 12).

Despite a large preclinical evidence, only a limited number of phytochemicals have progressed to clinical assessment which mainly demonstrate safety and modest biological activity rather than clear anticancer efficacy. Moreover, no clinical evidence currently supports the use of these compounds as immunotherapy sensitizers in melanoma or their ability to overcome the already established resistance mechanisms to the immune checkpoint blockade (Table 2).

Taken together, this comparative synthesis supports three main conclusions: (i) despite chemical diversity the phytocompounds show a mechanistic convergence, with effects on a shared network of pathways that are related to inflammation, immune suppression, survival signaling, and tumor–immune interaction; (ii) literature-based evidence suggests that the best supported role of these compounds is as biological modulators of the signaling and microenvironmental conditions that influence checkpoint response, rather than direct substitutes for checkpoint inhibitors; (iii) only a limited subset of compounds are backed by melanoma-specific immune-relevant findings sufficiently robust to warrant prioritization as candidate sensitizers.

## 7. Challenges and Limitations

This study reviewed the sensitizing role of phytocompounds in skin cancer immunotherapy, highlighting their ability to enhance the anticancer activity of conventional drugs but also to shape the TME to favor an antitumor immune response. However, there are limitations that hinder the transfer of preclinical results into clinical use, mainly related to the bioavailability and metabolism of phytocompounds, their toxicity and adverse effects and also the lack of standardized protocols which are required for the safety of oncological patients.

Polyphenols such as curcuma and resveratrol show unfavorable physicochemical and pharmacokinetic properties. For example, curcuma has low water solubility; thus, only a small amount reaches the blood and, subsequently, the tumor, thus achieving reduced oral bioavailability (<1%); additionally, curcuma is subjected to intense metabolism through glucuronidation and sulfation, therefore revealing a short plasma half-life. At physiological pH, the polyphenol is unstable, with approximately 78% of the compound rapidly degraded within 20 min. In addition, turmeric is sensitive to light and undergoes photoisomerization processes, which could reduce its therapeutic effects, with the occurrence of adverse effects such as mild hepatotoxicity. Its effective clinical use depends largely on the development of formulation strategies capable of overcoming these pharmacokinetic and biological barriers [281].

Other phytocompounds such as flavonoids, alkaloids, terpenoids and polysaccharides that are involved in the modulation of immune checkpoints in melanoma also display low solubility, rapid metabolism and, subsequently, low bioavailability. For example, quercetin exhibits low water solubility and short circulation time that reduce its bioavailability, which will be reflected in its low anticancer efficacy. In order to improve its bioavailability, cyclodextrins can be used to form inclusion complexes able to increase the phytocompound’s aqueous solubility [282].

Similarly, berberine, although exhibiting strong anticancer effects, has low stability in aqueous medium, reduced bioavailability and a short half-life; it is also involved in pharmacokinetic interactions due to its interference with enzymes employed in the metabolism of oncological drugs. To overcome these limitations, certain strategies were formulated to increase its water solubility, such as nanoparticles, liposome, and solid lipid nanoparticles, but also to delay its metabolic degradation through its co-administration with P-glycoprotein inhibitors or chemical modulation [283].

The low bioavailability is also an issue in the use of betulinic acid, a triterpenoid with high anticancer potential. Pharmacokinetic studies have shown that betulinic acid exhibits low solubility, preferential distribution into the adipose tissue, a large volume of distribution, and a half-life of 11–12 h, attributed to its lipophilicity as well as low systemic absorption. This issue becomes particularly important in immunotherapy, where specific concentrations are required to modulate the immune response against the tumor. In this context, betulinic acid–polyvinylpyrrolidone complexes, mucoadhesive microparticles, phospholipid-based nanosystems, and synthetic analogs are examples of strategies able to avoid such disadvantages. Furthermore, liposomes, transdermal systems, nanoemulsions and controlled release systems are new drug carriers that can enhance the selectivity, potency and pharmacokinetics of BA without compromising its anticancer potential [284].

For isothiocyanates, such as sulforaphane, the main challenge concerning their clinical application is the interindividual variation following their systemic administration, even at equivalent doses. Such variations are caused by physicochemical instability and rapid metabolism, particularly the rapid conjugation via the mercapturic acid pathway, leading to a short half-life and significant interindividual pharmacokinetic variability. Microencapsulation, enzyme-stabilized systems, and nanotechnology have been explored, and various preclinical studies have shown a clear increase in the bioavailability and cellular accumulation of sulforaphane compared to the free compound. However, the clinical utility of such approaches is still hampered by complexity and potential long-term toxicity [285,286].

The limitations of phytocompounds in terms of physicochemical and pharmacokinetic properties highlight not only the need for more detailed research including clinical trials, but also the need for standardized clinical protocols able to provide safe administration to oncological patients through correct dosage, reproducibility of studies and the evaluation of the efficacy and quality of phytocompounds.

## 8. Future Perspectives and Directions

Future investigations related to plant-derived compounds used as sensitizers for immunotherapy in melanoma should transition from general anticancer activity reports to validation of immunotherapy-related mechanisms. This review emphasizes that the main factors that drive immune checkpoint inhibition resistance are faulty antigen presentation, reduced IFN-γ/JAK/STAT signaling, immunosuppressive tumor microenvironments, T-cell exclusion, and compensatory inhibitory mechanisms. Thus, future endeavors should focus on selecting phytocompounds based on the potential to alter clinically significant resistance-inducing pathways, rather than only on their general cytotoxic or pro-apoptotic properties.

Stricter selection of potential drugs based on the quality of specific melanoma evidence is another crucial viewpoint. Despite the fact that numerous phytocompounds reviewed in this paper exhibit immunologically significant activity, only a few resemble the characteristics of a true immunotherapy sensitizer. Compounds active in melanoma models that can, at the same time, modify the tumor microenvironment, modulate checkpoint-related signaling, or increase tumor immunogenicity in this context should be submitted for further development. On the other hand, drugs that are only exhibiting indirect immunological effects or are inactive in melanoma models should be regarded with caution until their sensitizing role is more carefully verified.

Another important objective alludes to a clearer differentiation between immunomodulatory action and real checkpoint sensitization. Biologically relevant metrics for this case can be the regulation of PD-L1 expression, STAT3 signaling, macrophage polarization, or innate immune pathways. However, this will not inherently stand as a guarantee of enhanced efficacy in authorized immunotherapies. Further research should focus on how phytocompounds perform alongside anti-PD-1, anti-CTLA-4, or anti-LAG-3 therapies in melanoma models.

At the same time, their efficacy in overcoming JAK1/2 dysfunction, β2-microglobulin loss, impaired antigen presentation, or inadequate T-cell infiltration should also be evaluated. This degree of validation will be critical to determine if these chemicals can serve as authentic sensitizers or just broad adjuvant anticancer drugs.

Pharmacological optimization is also highlighted as a significant future direction. Poor water solubility, accelerated metabolism, low bioavailability, and limited tumor exposure are important obstacles towards translation that were constantly highlighted throughout this review. In this context, the enhancement of the clinical efficacy of these phytocompounds can be a result of both nanoformulation use, such as nanocarriers, lipid-based systems, polymeric nanoparticles, micelles, and chemical derivatization techniques. Future formulation studies must extend beyond just demonstrating increased delivery; they should determine if improved pharmacokinetics result in significant regulation of checkpoint-related processes and improved immunotherapeutic responses in vivo.

A promising approach is the development guided by biomarkers. Considering the pronounced genetic and immunological diversity of melanoma, forthcoming research ought to categorize experimental models and, ultimately, individuals based on immune phenotype, checkpoint profile, interferon signaling capability, and antigen-presentation status. This method can help us to better understand how certain phytocompounds perform in this biological context and facilitate the rational design of combination therapies.

The application of phytocompounds in melanoma immunotherapy requires standardization and, above all, clinical confirmation. Inadequate clinical data, the absence of standardized formulations and treatment methods, and uncertainty over appropriate dose and schedule constrain the existing evidence. Consequently, advancements in this field will rely on carefully designed, melanoma-specific preclinical investigations, standardized pharmaceutical models, biomarker-focused combinatorial strategies, and clinical trials. Currently, such compounds should be considered promising complementary therapies in melanoma immunotherapy, albeit not yet as clinically validated sensitizers.

## 9. Conclusions

Currently, vegetal compounds stand as a potential strategy to increase the efficacy of immunotherapy in melanoma, particularly due to their ability to act as cell sensitizers when associated with immune checkpoint inhibitors. A growing body of evidence suggests that phytocompounds are able to alter key mechanisms involved in tumor immune evasion, such as the TME, the immune checkpoint expression (e.g., PD-1/PD-L1 axis), and the T-cell activation, thus stimulating the antitumor immune responses and enhancing the overall therapeutic efficacy.

Their pleiotropic effects enable their interference with multiple signaling pathways simultaneously, which is especially useful in melanoma given its molecular heterogeneity and important acquired drug resistance. Moreover, recent data indicate that the combination of phytocompounds with immunotherapy, accompanied by the use of nanoparticulated delivery systems and personalized therapeutic strategies, may significantly improve drug bioavailability, reduce systemic toxicity, and optimize treatment efficacy.

Nonetheless, despite these promising preclinical results, the clinical application of phytocompounds is strongly challenged by their poor pharmacokinetic properties, low bioavailability, lack of standardized formulations, and insufficient clinical data. One may mitigate these limitations by designing smart drug delivery systems, clinical trials and standardized protocols that are essential for the successful clinical use of phytocompounds.

Therefore, phytocompounds can be regarded as potential adjuvants to immunotherapy in melanoma; however, additional studies are required to confirm their clinical performance and to establish their role within existing and forthcoming therapeutic strategies.

The use of phytocompounds as modulators of tumor immunity could shape the future of melanoma immunotherapy by intertwining natural bioactive substances with precision oncology. The future clinical value of these compounds will likely depend less on their intrinsic anticancer effects and more on their rational chemical optimization, targeted delivery, and proof of efficacy in models that bear established mechanisms of immunotherapy resistance.

## Figures and Tables

**Table 1:** Comparative evidence-based classification of plant-derived compounds as potential immunotherapy sensitizers in melanoma.

Chemical Class	Compound	Melanoma-Specific Evidence	Immune-Relevant Evidence in Melanoma	Direct Checkpoint-Related Relevance	TME Modulation	Main Dominant Mechanisms Reported	Evidence Tier
Flavonoids	Quercetin	High	High	Moderate	High	STAT3, CD47 axis, RIG-I/type I IFN, PD-L1-associated signaling, macrophage activation	Tier 1–2
	Luteolin	Moderate	Moderate	Low–moderate	Moderate	STAT3, APC activation, IL-12 signaling, CTL stimulation, anti-invasive activity	Tier 2
	Apigenin	Moderate	Moderate	Low	Moderate	Anti-inflammatory signaling, apoptosis/cell-cycle regulation, indirect immune support	Tier 2
	EGCG	Moderate	Moderate	Low–moderate	Moderate	Redox and inflammatory signaling, microenvironment-related effects	Tier 2
	Kaempferol	Moderate	Low–moderate	Low	Low–moderate	Oxidative stress and inflammatory signaling, tumor-intrinsic control	Tier 2
Polyphenols	Curcumin	Moderate	Moderate	Moderate	Moderate–high	NF-κB, STAT3, cytokine modulation, immune-context remodeling	Tier 2
	Resveratrol	Moderate	Moderate	Low–moderate	Moderate	Redox regulation, inflammatory signaling, adaptive immune-context effects	Tier 2
Isothiocyanates	Sulforaphane	Low	Low	Low	Low–moderate	Oxidative and inflammatory signaling modulation with limited melanoma-specific immunotherapy evidence	Tier 3
Triterpenes	Ursolic acid	Low–moderate	Low	Low	Moderate	Anti-inflammatory, anti-survival, indirect immune-context effects	Tier 3
	Betulinic acid and betulin	Variable	Low–moderate	Low	Moderate	Broad signaling and microenvironment-associated effects	Tier 2–3
Alkaloids	Matrine	Moderate	Moderate	Low	Low–moderate	PI3K/AKT/mTOR, Wnt/β-catenin, MAPK/ERK, PTEN upregulation, anti-invasive/apoptotic activity	Tier 2–3
	Berberine	High	High	Moderate–high	High	Macrophage repolarization, IL-6/STAT3/IL-10 axis inhibition, immunogenic cell death, dendritic-cell maturation, anti-PD-1/anti-CTLA-4 sensitization	Tier 1

The evidence tiers were assigned based on a comparison of the literature reviewed in this manuscript: Tier 1: melanoma-specific studies demonstrating immune-related effects and preclinical evidence of checkpoint-oriented sensitization or synergy. Tier 2: melanoma-specific tumor-intrinsic effects and indirect immune-related findings without a clear link to checkpoint sensitization. Tier 3: exploratory/weakly substantiated melanoma-specific sensitizing role or inadequately substantiated in melanoma-specific contexts.

**Table 2:** Clinical evidence supporting the translational relevance of plant-derived compounds in various cancers.

Compound	Clinical Setting	Cancer Type	Phase/Status	Key Findings	Reference
Curcumin	Oral administration (high-dose)	Colorectal/ GI cancers	Phase I–II (completed)	Safe up to 8–12 g/day; low bioavailability; tissue accumulation	[273]
Curcumin	Curcumin + chemotherapy (gemcitabine)	Pancreatic cancer	Phase II (completed)	Improved response in subset of patients; pharmacokinetic limitation persists	[273]
Curcumin (nano/delivery systems)	Exosome-based delivery	Colon cancer	Phase I (ongoing)	Designed to overcome bioavailability limitations	[274]
EGCG (Polyphenon E)	Green tea catechin formulation	Prostate cancer/premalignant lesions	Phase II (completed)	Reduced proliferation markers; modest clinical effect	[273]
EGCG	Dietary supplementation	Bladder cancer	Phase II (completed)	Reduced tumor proliferation biomarkers	[273]
Resveratrol	Oral supplementation	Colorectal cancer	Phase I–II (completed)	↓ Ki-67 proliferation index; metabolic effects	[275]
Resveratrol	Chemoprevention trials	Breast/multiple myeloma	Phase I–II (completed)	Epigenetic modulation; limited efficacy	[276]
Sulforaphane	Dietary/supplement	Prostate cancer	Phase II (completed)	Modulation of Nrf2 pathway and tumor biomarkers	[276]
Sulforaphane	Combination with chemotherapy	Breast cancer	Phase I–II (ongoing)	Evaluating protective and adjunct effects	[277]
Sulforaphane	Clinical evaluation	Multiple cancers (including melanoma)	Multiple trials	Limited number of trials; mainly biomarker endpoints	[276]
Quercetin + Curcumin	Combination therapy	Familial adenomatous polyposis	Pilot clinical study (completed)	↓ number and size of adenomas	[278]
Quercetin (alone)	Limited clinical data	Non-oncology primarily	Early-stage/sparse	No robust anticancer clinical evidence	[112]
Apigenin/ Luteolin	-	-	-	No clinical oncology trials identified	-
Curcumin + Ursolic Acid	Oral supplementation	Prostate cancer	Phase I	-	[279]
Ursolic acid	-	-	-	No validated clinical trials in cancer	
Betulinic acid	Topical treatment of dysplastic nevi	Precancerous lesions (melanoma risk)	Phase I/II (suspended)	Planned evaluation of safety and efficacy; study suspended due to funding; no published results	[280]
Betulinic acid	Topical application (ointment, various dosing regimens)	Cutaneous metastatic melanoma	Phase I (pilot, completed)	Evaluated safety, tolerability, apoptosis; no published outcome data; concentration not reported	[280]

↓—decrease. Note: The table summarizes the clinical data for the selected plant-derived compounds discussed in this review. The data supports their translational relevance in cancer-related settings but does not necessarily demonstrate direct immunotherapy-sensitizing effects or validated activity in melanoma.

## Data Availability

No new data were created or analyzed in this study. Data sharing is not applicable to this article.

## Abbreviations

The following abbreviations are used in this manuscript:

67LR	67 kDa laminin receptor
ABC	ATP-binding cassette
AKT	RAC (Rho family)-alpha serine/threonine-protein kinase
AMPK	AMP-activated protein kinase
APC	Antigen-presenting cell
BMI-1	B cell-specific Moloney murine leukemia virus integration site 1
BRAF	B-Rapidly Accelerated Fibrosarcoma
BTLA	B and T lymphocyte attenuator
CD	Cluster of differentiation
CTL	Cytotoxic T lymphocyte
CTLA-4	Cytotoxic T-Lymphocyte Antigen 4
DAMPs	Damage-associated molecular patterns
DR	Death receptor
EGCG	Epigallocatechin 3 gallate
EPR	Enhanced permeability and retention
ERK	Extracellular signal-regulated kinase
EZH2	Enhancer of zeste homolog 2
FAK	Focal adhesion kinase
FLIP	Cellular FLICE-like inhibitory protein
HDAC	Histone deacetylase
HMGB1	High-Mobility Group Box 1 protein
IFN	Interferon
IL	Interleukin
irAEs	Immune-related adverse events
IRF	Interferon regulatory factor
JAK	Janus kinase
LAG-3	Lymphocyte Activation Gene-3
MAPK	Mitogen-Activated Protein Kinase
MDSCs	Myeloid-derived suppressor cells
MHC I	Major histocompatibility complex I
MHC II	Major histocompatibility complex II
MMP	Matrix metalloproteinase
MPEG-PLA	Methoxy poly(ethylene glycol)-b-poly(L-lactide)
mTOR	Mechanistic target of rapamycin
NF-κB	Nuclear factor kappa-light-chain-enhancer of activated B cells
NK	Natural Killer
NRAS	Neuroblastoma RAS viral oncogene homolog
Nrf2	Nuclear factor erythroid 2-related factor 2
NSCLC	Non-small cell lung cancer
NQO1	NAD(P)H quinone oxidoreductase 1
OCT4	Octamer-binding transcription factor 4
ORR	Objective response rate
OS	Overall survival
PDK1	Phosphoinositide-dependent protein kinase-1
PD-L1	Programmed Death-Ligand 1
PFS	Progression-free survival PFS
PI3K	Phosphoinositide 3-Kinase
PTEN	Phosphatase and tensin homolog
RARβ	Retinoic acid receptor beta
RCC	Renal cell carcinoma
RIG-I	Retinoic acid-inducible gene I
ROS	Reactive oxygen species
SHP	Src homology region 2-containing phosphatase-2
SIRPα	Signal Regulatory Protein alpha
SOX	SRY-box transcription factor
STAT	Signal transducer and activator of transcription
TAMs	Tumor-associated macrophages
TCR	T-cell receptor
TGF-β	Transforming Growth Factor-β
TIGIT	T-cell immunoreceptor with immunoglobulin and ITIM domain
TIM-3	T-cell immunoglobulin and mucin domain-containing protein 3
TME	Tumor microenvironment
TRAIL	Tumor Necrosis Factor-Related Apoptosis-Inducing Ligand
Treg	Regulatory T cells
VEGF	Vascular Endothelial Growth Factor
VISTA	V-domain Ig suppressor of T-cell activation
WBC	White blood cell
ZAP-70	Zeta-chain-associated protein kinase 70
